# *In planta* expression of nanobody-based designer chicken antibodies targeting *Campylobacter*

**DOI:** 10.1371/journal.pone.0204222

**Published:** 2018-09-27

**Authors:** Charlotte Vanmarsenille, Jelle Elseviers, Charlotte Yvanoff, Gholamreza Hassanzadeh-Ghassabeh, Gabriela Garcia Rodriguez, Edo Martens, Ann Depicker, An Martel, Freddy Haesebrouck, Frank Pasmans, Jean-Pierre Hernalsteens, Henri De Greve

**Affiliations:** 1 VIB-VUB Center for Structural Biology, Brussels, Belgium; 2 Structural Biology Brussels, Vrije Universiteit Brussel, Brussels, Belgium; 3 Viral Genetics, Vrije Universiteit Brussel, Brussels, Belgium; 4 Department of Pathology, Bacteriology and Avian Diseases, Faculty of Veterinary Medicine, Ghent University, Merelbeke, Belgium; 5 VIB Nanobody Core, Vrije Universiteit Brussel, Brussels, Belgium; 6 ARG VUB-UGent NanoMicrobiology, IJRG VUB-EPFL BioNanotechnology & NanoMedicine, Structural Biology Brussels, Vrije Universiteit Brussel, Brussels, Belgium; 7 Department of Plant Biotechnology and Bioinformatics, Ghent University, Ghent, Belgium; 8 VIB Center for Plant Systems Biology, Ghent, Belgium; Instituto Butantan, BRAZIL

## Abstract

Campylobacteriosis is a widespread infectious disease, leading to a major health and economic burden. Chickens are considered as the most common infection source for humans. *Campylobacter* mainly multiplies in the mucus layer of their caeca. No effective control measures are currently available, but passive immunisation of chickens with pathogen-specific maternal IgY antibodies, present in egg yolk of immunised chickens, reduces *Campylobacter* colonisation. To explore this strategy further, anti-*Campylobacter* nanobodies, directed against the flagella and major outer membrane proteins, were fused to the constant domains of chicken IgA and IgY, combining the benefits of nanobodies and the effector functions of the Fc-domains. The designer chimeric antibodies were effectively produced in leaves of *Nicotiana benthamiana* and seeds of *Arabidopsis thaliana*. Stable expression of the chimeric antibodies in seeds resulted in production levels between 1% and 8% of the total soluble protein. These *in planta* produced antibodies do not only bind to their purified antigens but also to *Campylobacter* bacterial cells. In addition, the anti-flagellin chimeric antibodies are reducing the motility of *Campylobacter* bacteria. These antibody-containing *Arabidopsis* seeds can be tested for oral passive immunisation of chickens and, if effective, the chimeric antibodies can be produced in crop seeds.

## Introduction

The incidence of campylobacteriosis has been increasing in the last years in both the developed and the developing world [1,2]. The majority of the human infections are caused by *Campylobacter jejuni* and *Campylobacter coli*, and broilers, where *Campylobacter* mainly colonises the intestinal tract [3], are the most common source of infection in industrialised countries. Symptoms of *Campylobacter* infection are diarrhoea, headache and fever and are mostly self-limiting [4,5]. In some cases, the infection has more severe consequences, like other gastrointestinal illnesses such as inflammatory bowel disease, colorectal cancer and the autoimmune diseases Guillain-Barré and Miller Fisher [1]. Colonisation of broilers by *Campylobacter* is typically asymptomatic [6]. During the first two to three weeks after hatching, broilers are protected against *Campylobacter* colonisation by the presence of *Campylobacter-*specific maternal antibodies. Sahin *et al*. [7] demonstrated the contribution of maternal antibodies to the absence of *Campylobacter* bacteria in chicks. Protection ultimately ceased, which led to a rapid spread of *Campylobacter* within the broiler flock by horizontal transmission, by the faecal-oral route, through feed and water [8]. Effective transmission causes a high prevalence of *Campylobacter* in broilers at slaughter age, typically at an age of six to seven weeks, leading to a high risk of carcass contamination [9]. Successful protection of broilers against *Campylobacter* is needed and passive immunisation-based strategies are promising for colonisation control [7,10]. Reduction of the *Campylobacter* load in the chicken caecum should result in a decrease of the number of human infections [11]. Because no efficient control strategies are available, the potential of novel methods needs more thorough exploration [9,12]. The use of antibiotics in animal feed to control colonisation leads to the rise of resistant strains [13]. The young age at which broilers are slaughtered and the time needed to induce antibody production in case of vaccination complicate the development of an effective vaccine [7,14]. However, previous studies have shown the potential of passive immunisation. Reduction of the *C*. *jejuni* count in the caeca of infected chickens was observed after feeding egg yolks, rich in IgY, from *C*. *jejuni-*immunised hens [10,15].

Nanobodies (Nb), the antigen-binding domains of camelid heavy-chain antibodies, possess several advantageous characteristics, which make their use as diagnostics and therapeutics interesting. Nanobodies show high affinity, specificity and stability and they can remain functional under harsh chemical and thermal conditions [16]. Because of their extended complementarity determining region 3 (CDR3), they have the capability of binding to buried epitopes and recognising a broader range of epitopes on the antigen [17]. However, single nanobodies are monovalent and are rapidly cleared from the host. These disadvantages can be circumvented by the fusion of nanobodies to the Fc-domain of an immunoglobulin, combining the benefits of a nanobody with an effector function. This leads to multiple valences, which makes agglutination of the bacteria possible [18]. Fusion of the nanobodies to the Fc-domain will lead to an extended half-life *in vivo*, by an increase in size and interaction with Fc-receptors [19,20] and could result in lowering the doses required for therapeutic treatment [21]. Another advantage of the Nb-Fc constructs is that the fusions are encoded by only one gene. In case of classical antibodies, the genes encoding the light and the heavy chain must be co-expressed [22].

Virdi *et al*. [23] successfully used nanobodies, generated against the F4-fimbriae of enterotoxigenic *E*. *coli* (ETEC), fused to the Fc-domain of pig IgA, for the passive vaccination of piglets against ETEC infections. The chimeric antibodies were expressed in *Arabidopsis thaliana* seeds and administered to the piglets via their feed. Bacterial colonisation was significantly reduced after challenge with an F4-positive ETEC strain. Expression in plants of recombinant proteins is cost-effective and can easily be scaled-up. Correct folding and the desired post-translational modifications are commonly achieved [24]. Production of recombinant proteins in seeds allows stable storage for long periods [25]. Other advantages are the ease of oral administration of the seeds via the animal feed and the potential protection of the chimeric antibodies against digestion by proteases in the stomach [26].

In this study, a strategy similar to that used by Virdi *et al*. [23] was followed to produce antibodies that can be applied for passive immunisation of broilers against *Campylobacter*, by means of chimeric antibodies expressed in seeds of *A*. *thaliana*. *Campylobacter-*specific nanobodies directed against the major outer membrane protein (MOMP) [27] and flagella, both important virulence factors, were selected. The MOMP is crucial for the viability of the bacterial cells and is involved in adhesion to intestinal cells, whereas flagella are essential for motility, colonisation and pathogenesis [28–30]. These nanobodies were fused to the constant domains of chicken immunoglobulins (Ig). The major serum immunoglobulin of chickens is IgY, whereas the antibody most abundantly found in the intestinal tract is IgA [31,32]. In this study, the construction and expression of chimeric IgY and IgA antibodies is described. These were transiently expressed in *Nicotiana benthamiana* leaves and stably in *A*. *thaliana* seeds, under the control of the seed-specific β-phaseolin promoter. We show that these chimeric antibodies recognise not only their respective target antigens but also the *Campylobacter* bacteria.

## Materials and methods

### Growth conditions of *Campylobacter*

*C*. *jejuni* KC40 [33] was grown on Nutrient Broth Nr.2 solidified with 1.5% agar (NB2, CM0067; Thermo Fisher Scientific) under microaerobic conditions (Oxoid CampyGen, Thermo Fisher Scientific) for 48 hours at 42°C.

### Purification of MOMP and flagella

MOMP was purified from a total membrane extract, essentially as described by Hobb *et al*. and Bolla *et al*. [34,35]. Further purification was performed by anion-exchange chromatography, using a Resource Q column (GE Healthcare Life Sciences). The latter was equilibrated with 20 mM sodium phosphate buffer pH 6.0 supplemented with 0.6% poly(ethylene glycol) octyl ether (octyl-POE) and the extracted proteins were loaded on the equilibrated column. A linear gradient to 1 M NaCl was used for elution. The eluted fractions were analysed by SDS-PAGE and Coomassie blue staining. Pure fractions were dialysed against 20 mM sodium phosphate buffer pH 7.6 with 0.6% octyl-POE. Flagellins were isolated from *C*. *jejuni* KC40 (S1 File), as described by Logan and Trust [36].

### Isolation of nanobodies targeting *Campylobacter*

Nanobodies against the MOMP (Nb5 and Nb23) [27] and flagella (Nb2Flag8, Nb2Flag24 and Nb2Flag67) of *Campylobacter* were obtained from a nanobody library by phage display [27,37] (S2 File). A C-terminal histidine-tag was added to the nanobodies, by subcloning the nanobody-encoding genes in the expression vector pHEN6c, a derivative of the pHEN6 vector [38]. The In-Fusion HD Cloning Kit (Takara Bio USA, Inc) was used for the introduction of the nanobodies in the pHEN6c vector, digested with PstI and BstEII. The nanobody-encoding sequences were amplified with the primers IF-NB1 (5’-TGGCCCAGGTGCAGCTGCAGGAGTCTGGAG-3’) and IF-NB2 (5’-TGAGGAGACGGTGACCTGGGTCC-3’). The reaction mix was transformed into CaCl_2_-competent *E*. *coli* DH5α [39] and transformants were selected on LB-agar plates with 100 μg/ml carbenicillin. The expression vector from positive transformants was introduced in *E*. *coli* WK6 for expressing the nanobodies. The bacterial cells were grown in the presence of carbenicillin (100 μg/ml) at 37°C. When an OD_660 nm_ 0.6–0.8 was reached, the addition of 1 mM isopropyl β-D-1 thiogalactopyranoside (Thermo Fisher Scientific) led to the expression of the nanobodies. After overnight incubation, a periplasmic extract was prepared and the His-tagged nanobodies were further purified by nickel-affinity chromatography. Therefore, the sample in 20 mM Tris-HCl, 1M NaCl, pH 8.0 was loaded on a HisTrap HP column (GE Healthcare Life Sciences) and nanobodies were eluted using a linear gradient to 1 M imidazole. Finally, pure fractions were stored at -20°C in phosphate-buffered saline (PBS).

### Thermal and pH stability of nanobodies

The stability of the nanobodies under different pH conditions was confirmed using SYPRO orange dye with the Thermofluor assay [40]. The details of the experimental approach are extensively described in S3 File.

### Fusion of *Campylobacter*-specific nanobodies to the constant domain of chicken antibodies

**Fig 1:**
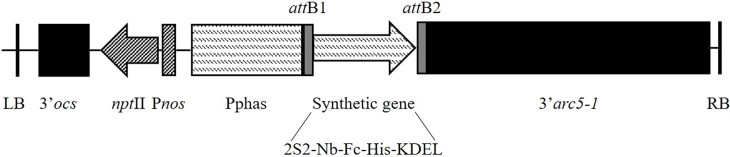
T-DNA construct used for the expression of the chimeric antibodies. (LB) left border, (3’OCS) octopine synthase terminator, (*npt*II) neomycin phosphotransferase II gene, (P*nos*) nopaline synthase promoter, (Pphas) β-phaseolin promoter, (*att*B1 & *att*B2) attachment sites for Gateway recombination, (2S2) signal peptide of the 2S2 seed storage protein, (Nb-Fc) Fusion of anti-*Campylobacter* nanobody to chicken IgA or IgY, (His) histidine-tag, (KDEL) endoplasmic retention peptide, (3’*arc5-1*) arcelin terminator and (RB) right border.

Synthetic genes were designed for the fusion of Nb5 to the Fc-domain of chicken IgA [41] and the constant domains of chicken IgY [42], in which the codon usage is optimized for expression in plants (S4 File). The nanobody-encoding sequence was preceded by the signal sequence of the seed storage protein 2S2 of *A*. *thaliana* for targeting to the endoplasmic reticulum. At the C-terminus of the synthetic gene, a histidine-tag (His) and a KDEL signal were added (Fig 1). The latter is necessary for retention in the lumen of the endoplasmic reticulum. The attachment sites (*attB*1 and *attB*2), at both ends of the synthetic gene, make Gateway recombination possible. A BP reaction was used for cloning of the synthetic gene in the pDONR221 Gateway donor vector (Gateway Technology). The other nanobodies were introduced in the entry clone, encoding the Nb5-Fc fusion, by exchanging Nb5. The latter was removed by restriction of the entry clone with PstI and BstEII. For the amplification of the nanobody-encoding sequences, the primers T-NbS1 and T-NbS2 were used (S4 File). In-Fusion cloning was subsequently applied for the insertion of the sequences encoding Nb23, also directed against the MOMP, and V1, against the F4-fimbriae of enterotoxigenic *E*. *coli* [23], that was used as a control. The anti-flagellin nanobodies (Nb2Flag8, Nb2Flag24 and Nb2Flag67) were similarly fused to the Fc-domain of IgA.

### Transient expression in *N*. *benthamiana* leaves

For transient expression, the plasmid pEAQ-*HT*-DEST1 (43) was used. The entry clones were introduced in this vector using an LR reaction (Gateway Technology). The resulting clones (Table 1) were transformed via electroporation in *A*. *tumefaciens* LBA4404 [44,45]. The *A*. *tumefaciens* strains were subsequently used for the infiltration of *N*. *benthamiana* leaves [46]. One week later, the infiltrated areas of the leaves were harvested and protein extracts were prepared as described [46].

**Table 1:** The pEAQ-*HT*-DEST1 and pPhasGW expression plasmids encoding nanobodies fused to the constant domains of IgA or IgY.

Plasmid	Characteristics
pEAQ-*HT*	pEAQspecialK with CPMV-*HT* cassette [43]
pEAQ-*HT-*DEST1	Gateway-compatible pEAQ-*HT* destination vector [43]
pGV5689	pEAQ-*HT*-DEST1 + Nb5-IgY
pGV5679	pEAQ-*HT*-DEST1 + Nb5-IgA
pGV5923	pEAQ-*HT*-DEST1 + Nb2Flag8-IgA
pGV5925	pEAQ-*HT*-DEST1 + Nb2Flag24-IgA
pGV5927	pEAQ-*HT*-DEST1 + Nb2Flag67-IgA
pPhasGW	Gateway-compatible vector [47]
pGV5774	pPhasGW + V1-IgY
pGV5768	pPhasGW + V1-IgA
pGV5772	pPhasGW + Nb5-IgY
pGV5778	pPhasGW + Nb5-IgA
pGV5776	pPhasGW + Nb23-IgY
pGV5770	pPhasGW + Nb23-IgA

### Stable expression in *A*. *thaliana* seeds

For stable expression in seeds, the entry clones were inserted in the Gateway-compatible pPhasGW vector (Table 1), via an LR reaction. In the T-DNA, the chimeric genes are under the control of the strong seed-specific β-phaseolin promoter. The T-DNA also encodes the kanamycin resistance gene *npt*II, allowing selection of transformed plants (Fig 1). The resulting clones were subsequently transformed via electroporation [45] in *A*. *tumefaciens* C58C1 Rif^R^ (pMP90) [48]. *A*. *thaliana* Columbia (Col-0) plants were transformed with *A*. *tumefaciens* C58C1 Rif^R^ (pMP90) harbouring the corresponding constructs in the pPhasGW vector (pGV5768, pGV5774, pGV5772, pGV5778, pGV5770 and pGV5776) (Table 1) via the floral dip method [49].

Samples (about 10 mg) of the obtained T1 seeds were surface-sterilized by washing with 70% ethanol for 2 minutes, followed by incubation in commercial bleach solution (10°Chl) supplemented with 0.1% Tween-20 for 15 minutes and washed three times with sterile water. Four ml 0.3% agar was added to the sterile seeds and these were plated on 20 ml K1 medium [46] supplemented with Timentin (160 μg/ml), nystatin (50 μg/ml) and kanamycin (50 μg/ml) in 9.4-cm Petri dishes sealed with gas-permeable tape. After storage at 4°C for 48 hours, the dishes were incubated at 24°C under a 16-hours light / 8-hours dark cycle. After 3 weeks, the resistant seedlings were transferred to commercial potting mix in the greenhouse for seed production by spontaneous self-pollination.

From the kanamycin-resistant T1 plants, T2 seeds were harvested and seed extracts were prepared. For each chimeric antibody construct, seeds of twenty plants were tested. As a negative control, protein extractions were performed on seeds of untransformed *A*. *thaliana* Columbia plants.

### Protein extraction from *A*. *thaliana* seeds

For protein extraction, 10 mg seeds were weighed in a 2 ml microcentrifuge tube and two 4 mm stainless steel balls were added. The tubes were frozen in liquid nitrogen and the seeds were pulverised using the Retsch Mixer Mill MM 300 during 2 minutes at 25 Hz. The crushed seeds were resuspended in extraction buffer (50 mM NaH_2_PO_4_ pH 7.8, 300 mM NaCl, 10 mM EDTA, 0.1% Tween-20) supplemented with cOmplete Protease Inhibitor Cocktail (Roche Diagnostics) in a 1:100 ratio (mg seeds/μl extraction buffer). The suspension was centrifuged at 20000 g for 5 minutes at 4°C and the supernatant was mixed with glycerol (final concentration 20% v/v) and stored at -20°C.

### ELISA

Seed extracts of independent *A*. *thaliana* transformants, 100 μl of a 1/20 dilution, were coated in 96-well plates in coating buffer (150 mM Na_2_CO_3_, 46 mM NaHCO_3_). Overnight incubation at 4°C was followed by five wash steps with PBS + 0.05% Tween-20. Then 200 μl 5% bovine serum albumin (BSA) was added to each well and incubated for 2 hours at room temperature. Subsequently, the wells were washed five times and 100 μl goat anti-Chicken IgA (1/5000) or rabbit anti-Chicken IgY (1/1250), both conjugated to HRP (horseradish peroxidase) (Thermo Fischer Scientific), was added to each well and incubated for 1 hour at room temperature. The ELISA was developed by addition of TMB Substrate Solution (Thermo Fisher Scientific). The reaction was stopped using 0.16 M H_2_SO_4_ (100 μl/well) and the signal was read at 450 nm.

For the interaction assay of the nanobodies or the chimeric antibodies with their antigen, purified MOMP or flagellins were coated in the 96-well plates at a concentration of 1 μg/ml. Whole-cell ELISA was used to assess their interaction with *C*. *jejuni* KC40 bacteria. Bacteria were grown on NB2 medium for 48 hours and harvested with PBS. The cells were pelleted at 3600 g for 15 minutes, resuspended in PBS and fixed by the addition of 2.5% (v/v, final concentration) of methanol-stabilized 37% formaldehyde solution (Merck) and incubation at 42°C for 100 minutes. Then the bacterial cells were pelleted, resuspended in coating buffer and the OD_660_ was adjusted to 0.3 before coating. After blocking with 200 μl 5% BSA and washing with PBS + 0.05% Tween-20, His-tagged nanobodies (50 μg/ml) or chimeric antibodies were added. Bound His-tagged nanobodies were detected using mouse anti-histidine tag monoclonal antibody (1/1000) (AbD Serotec) and goat anti-mouse IgG conjugated to alkaline phosphatase (AP) (1/5000) (Sigma-Aldrich). The ELISA was developed by adding 100 μl of developer solution, 2 mg/ml para-nitrophenyl phosphate (p-NPP) in ELISA buffer (100 mM Tris-HCl, pH 9.5, 5 mM MgCl_2_, 100 mM NaCl), and the OD was read at 405 nm. For the detection of bound IgA or IgY chimeric antibodies, goat anti-Chicken IgA (1/5000) or goat anti-Chicken IgY (1/1250), both conjugated to HRP (Abcam), were added.

### Western blotting

Protein extracts (200 μl) were TCA-precipitated and resuspended in 20 μl deionised water and 20 μl 2X loading buffer. The TCA-precipitated extracts were boiled and loaded on a 12.5% acrylamide gel. After running, the gel was stained with Coomassie blue dye or the proteins were transferred to a polyvinylidene difluoride (PVDF) membrane, which was previously activated with methanol. The PVDF membrane was then washed five times with PBS + 0.2% Triton X-100 and incubated with blocking buffer (PBS + 10% milk powder) for 1 hour at 4°C. The presence of the chimeric antibodies was subsequently detected with mouse anti-histidine tag monoclonal antibody (1/1000) (AbD Serotec) and anti-mouse IgG conjugated to HRP (1/5000). The western blot was developed using the Pierce ECL Western Blotting Substrate. Images were made with the Molecular Imager ChemiDoc XRS+ (Biorad).

Protein-protein interactions were detected by western blotting on native purified antigens (5 μM). The samples were loaded on a 12.5% acrylamide gel in loading buffer without DTT and running buffer composed of 14.4 g glycine, 3.03 g Tris and 0.250 g SDS per litre. Electrophoresis was followed by staining with Coomassie blue dye or transfer to a PVDF membrane. A 1/20 dilution of the extracts was used to examine the interaction with their antigen. Subsequently, the western blot was developed using goat anti-IgA (1/5000) or goat anti-IgY (1/1250) conjugated to HRP (Abcam).

### Immunofluorescence microscopy

*C*. *jejuni* bacteria were grown microaerobically on NB2 agar plates. After two days, the cells were harvested with NB2 medium and fixed by the addition of 2.5% (v/v, final concentration) of methanol-stabilized 37% formaldehyde solution (Merck). Afterwards, the cells were pelleted and resuspended in PBS. Of the fixed bacterial cells, 10 μl was spotted on 0.1% poly-L-lysine-treated glass slides and incubated for 15 minutes. The slides were treated with 5% BSA for 15 minutes and then 30 μl of anti-*Campylobacter* nanobodies (50 μg/ml) or undiluted seed extract were added. After 1 hour incubation at room temperature, the bound His-tagged nanobodies were detected using a mouse anti-histidine tag monoclonal antibody (1/200) (AbD Serotec) and anti-mouse IgG conjugated to Alexa Fluor 488 (1/250) (Thermo Fisher Scientific).

The chimeric antibodies in the seed extracts were detected using goat anti-IgA (1/200) or goat anti-IgY (1/200) (Abcam) and anti-goat IgG conjugated to Alexa Fluor 488 (1/250) (Abcam). The antibodies were each time incubated for 30 minutes at room temperature. After each step, the glass slides were washed with PBS. The nanobody V1 was used as a negative control (23). Images were acquired using an inverted epifluorescence microscope (Nikon Eclipse TE2000-U) (Objective = 100x) and a FITC filter block.

### Motility assay

A motility assay was performed with the chimeric antibodies. Seed extract containing anti-flagellin chimeric antibodies (± 50 μg/ml) (S1 Table) was pre-incubated with equal volumes of *C*. *jejuni* KC40 (OD_660_ 0.3) for 1 hour at room temperature. Of the suspension, 10 μl was spotted on an NB2 plate with 0.4% agar and incubated at 42°C under microaerobic conditions. After 24h, 48h and 72h, the diameter of the bacterial migration zone was measured.

## Results

### Design of chimeric genes encoding MOMP-recognising antibodies

Nb5 and Nb23 are directed against the MOMP and in a previous study it was shown that these nanobodies have a broad host specificity, interacting with multiple *Campylobacter* strains [27]. The interaction of the nanobodies with different isolates was confirmed by immunofluorescence microscopy (S1 Fig). The latter makes them an interesting choice for the development of chimeric antibodies. Bivalent nanobody constructs were obtained by the fusion of these nanobodies with the codon-optimised Fc-domain of IgA or the constant domains of IgY. Similar constructs, with the anti-*E*. *coli* nanobody V1, were made as controls. This resulted in six constructs: Nb5-IgA, Nb5-IgY, Nb23-IgA, Nb23-IgY, V1-IgA and V1-IgY.

### MOMP-specific chimeric antibodies are transiently expressed in leaves

**Fig 2:**
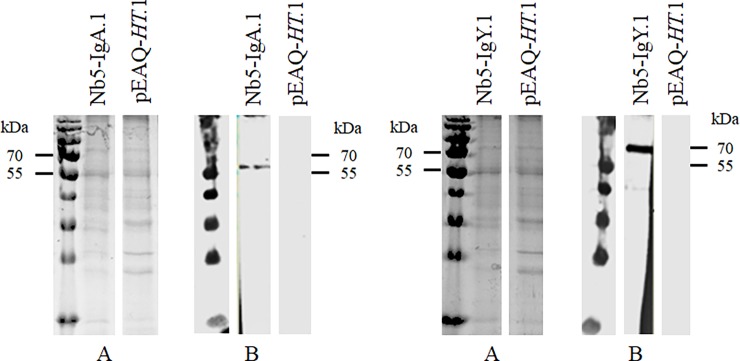
Transient expression of chimeric antibodies carrying Nb5 in leaves of *N*. *benthamiana*. Protein extracts were analysed using (A) SDS-PAGE stained with Coomassie blue dye and (B) western blot developed with mouse anti-histidine tag monoclonal antibody. Extracts of *N*. *benthamiana* leaves transformed with Nb5-IgA and Nb5-IgY fusion constructs were tested. Leaves infiltrated with *A*. *tumefaciens* harbouring the vector pEAQ*-HT* were used as negative control.

Transient expression in leaves is an excellent method for rapidly assessing whether a gene is expressed and to obtain a small quantity of recombinant protein. For this aim, the Nb5 fusion constructs were transiently expressed in leaves of *N*. *benthamiana* by infiltration with *A*. *tumefaciens* LBA4404 harbouring the plasmids pGV5679 and pGV5689 (Table 1). As a negative control, leaves were infiltrated with *A*. *tumefaciens* LBA4404 containing the pEAQ-*HT* plasmid. Extracts of the infiltrated leaves were screened for the presence of Nb5-Fc fusion proteins. After SDS-PAGE and Coomassie Blue staining, no clear difference in protein bands was observed between these extracts and the negative control (Fig 2A). Based on the amino acid sequence, molecular weights of approximately 50 and 63 kDa were expected for the IgA and IgY fusion constructs, respectively. Western blot, using an anti-histidine tag monoclonal antibody, showed bands of approximately 60 kDa and 70 kDa, for the IgA and IgY fusion constructs, respectively (Fig 2B). Glycosylation is probably responsible for the higher molecular weight.

### Stable expression of chimeric antibodies in seeds

The previous results show the feasibility of the expression of the chicken antibody constructs in plants. However, for further work, larger quantities are required. Therefore, the chimeric genes, encoding V1-IgA, V1-IgY, Nb5-IgA, Nb5-IgY, Nb23-IgA and Nb23-IgY, were stably expressed in *A*. *thaliana* seeds under the control of the β-phaseolin promoter.

**Fig 3:**
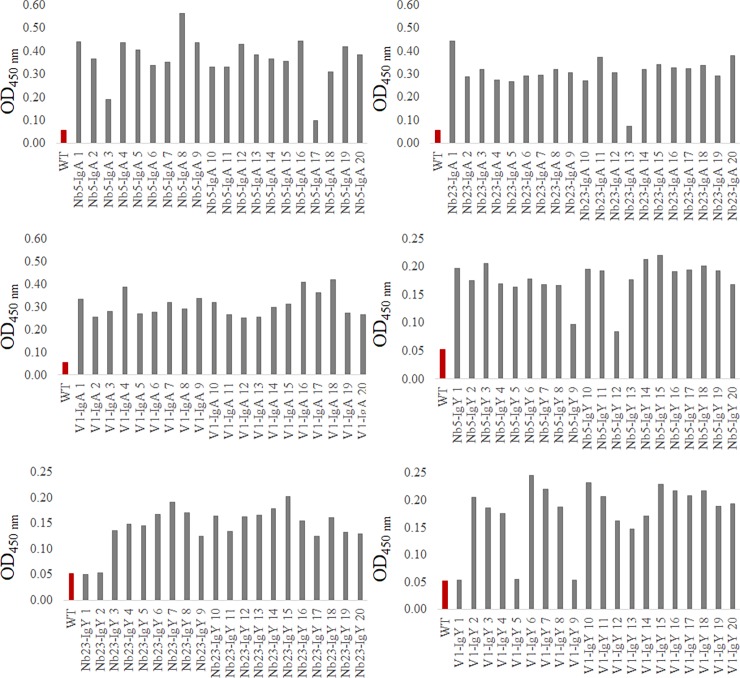
Expression of chimeric antibodies in transgenic *A*. *thaliana* seeds. Extracts of T2 seeds were coated and analysed by ELISA. The presence of chimeric antibodies in the extracts was tested, using anti-chicken IgA or IgY conjugated to HRP. As a negative control, extract of wild-type *A*. *thaliana* seeds was used. The results of seed extracts from *A*. *thaliana* plants transformed with Nb5-IgA, Nb23-IgA, V1-IgA, Nb5-IgY, Nb23-IgY and V1-IgY are shown.

The expression of Nb-IgA and Nb-IgY constructs in T2 seeds was tested via ELISA, using polyclonal anti-IgA or anti-IgY antibodies, respectively (Fig 3). Except for a few transformants, all extracts were positive in ELISA. The variations in expression levels between most of the positive seed extracts were small. Positive transformants were chosen for further experiments.

**Fig 4:**
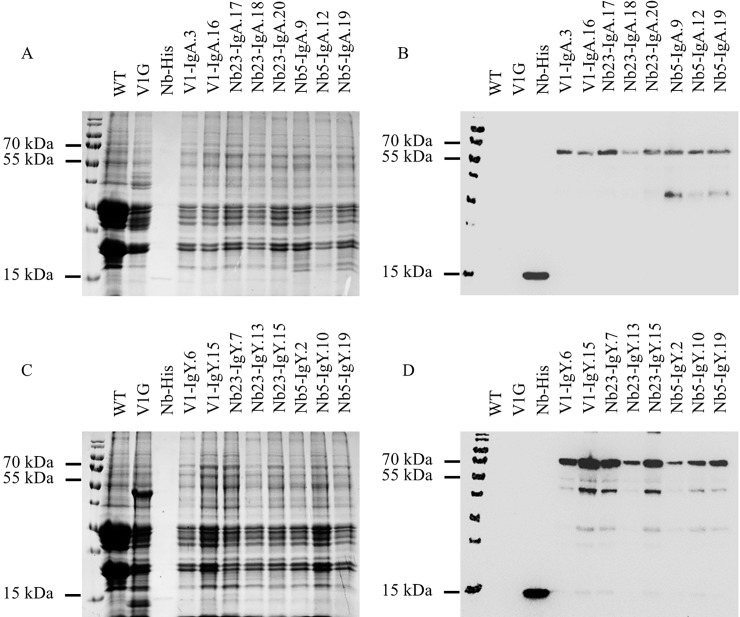
Screening for the expression of chimeric antibodies in *A*. *thaliana* seeds using SDS-PAGE (A, C) and western blot (B, D). The expression of chimeric antibodies in seeds was confirmed for extracts positive in ELISA. Western blots were developed with a mouse anti-histidine tag monoclonal antibody and a goat anti-mouse antibody conjugated to HRP. As negative controls, extract of wild-type seeds and the V1G chimeric antibody lacking the His-tag [23] were used. A His-tagged nanobody was used as a positive control, resulting in a protein band with a molecular weight of approximately 15 kDa. (A) IgA chimeric antibodies with V1, Nb23 and Nb5, (C) IgY chimeric antibodies with V1, Nb23 and Nb5.

Seed extracts were analysed by SDS-PAGE and western blot, to confirm expression (Fig 4). Like in the results of the transient expression, no clear differences in band pattern were observed after Coomassie Blue staining, between the extract of wild-type *A*. *thaliana* seeds and the ones transformed with any of the six chimeric antibody constructs. However, the western blot with mouse anti-histidine tag monoclonal antibody clearly confirmed the presence of the chimeric antibodies in the seeds of transformed plants. Besides the intact protein, lower molecular weight bands were also observed, presumably as a consequence of proteolytic activity. An additional negative control, used in this western blot, was the V1G chimeric antibody, lacking the His-tag, fused with the Fc of porcine IgG, directed against F4-positive enterotoxigenic *E*. *coli* [23]. As a positive control, a His-tagged nanobody was used, that produced a protein band with a molecular weight of approximately 15 kDa.

**Fig 5:**
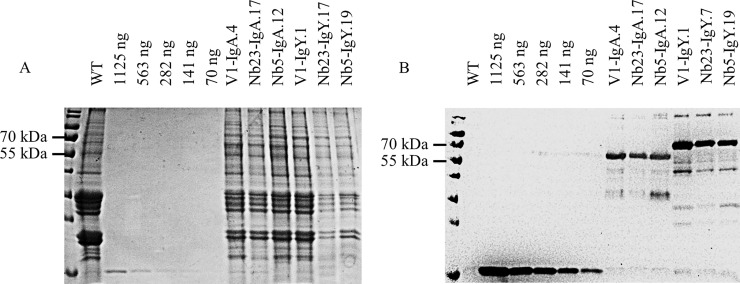
Determination of the concentration of chimeric antibodies present in seed extracts. Intensities on (A) Coomassie blue stained SDS-PAGE and (B) western blot of protein bands corresponding to a His-tagged nanobody and the ones corresponding to chimeric antibodies were compared. A serial dilution of a His-tagged nanobody with a concentration of 1.2 mg/ml was made. As a negative control, seed extract of wild-type A. thaliana plants was used. The western blot was developed with a mouse anti-histidine tag monoclonal antibody and goat anti-mouse IgG conjugated to HRP.

The use of an anti-His antibody, for the screening of IgA as well as IgY constructs makes it possible to compare the quantity of chimeric antibodies present in the extracts. For this aim, an SDS-PAGE and a western blot, developed with a mouse anti-histidine tag monoclonal antibody were used (Fig 5). Concentrations were determined by comparison of the intensity of the band of a His-tagged nanobody, having a known concentration (1.2 mg/ml), with that of the band corresponding to chimeric antibodies, using the ImageJ program (https://imagej.nih.gov/ij/). On this basis, expression levels between 1 and 8% of the total soluble proteins (TSP) were estimated for the four chimeric antibodies (Table 2).

**Table 2:** Chimeric antibody concentration in extracts of transformed *A*. *thaliana* seeds.

Chimeric antibody	Concentration (μg/ml)	μg/mg seed	%TSP*
Nb5-IgA	24.3	2.4	1.2
Nb23-IgA	21.6	2.2	1.1
Nb5-IgY	92.7	9.3	4.6
Nb23-IgY	156.4	15.6	7.8

*TSP: total soluble protein.

### In seed produced chimeric antibodies bind native MOMP and *C*. *jejuni* bacteria

**Fig 6:**
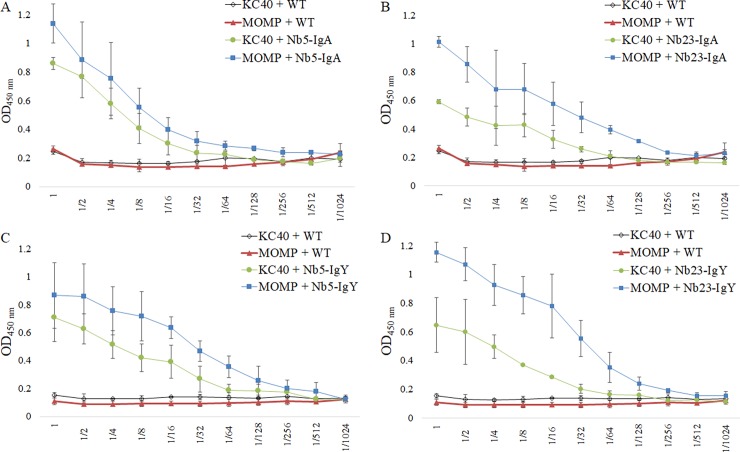
Binding of chimeric antibodies to *C*. *jejuni* KC40 bacteria and purified MOMP. KC40 bacteria and MOMP (1 *μ*g/ml) were coated in an ELISA plate. Subsequently, the interaction of twofold serial dilutions of the seed extracts was assessed. Therefore, anti-IgA and anti-IgY antibodies, conjugated to HRP, were used for the detection of chimeric antibodies, bound to the bacteria or to the purified native MOMP antigen. As a negative control, the binding of extracts of wild-type *A*. *thaliana* seeds with the bacteria and MOMP was measured. (A) Nb5-IgA, (B) Nb23-IgA, (C) Nb5-IgY and (D) Nb23-IgY. The error bars correspond to the standard deviations.

**Fig 7:**
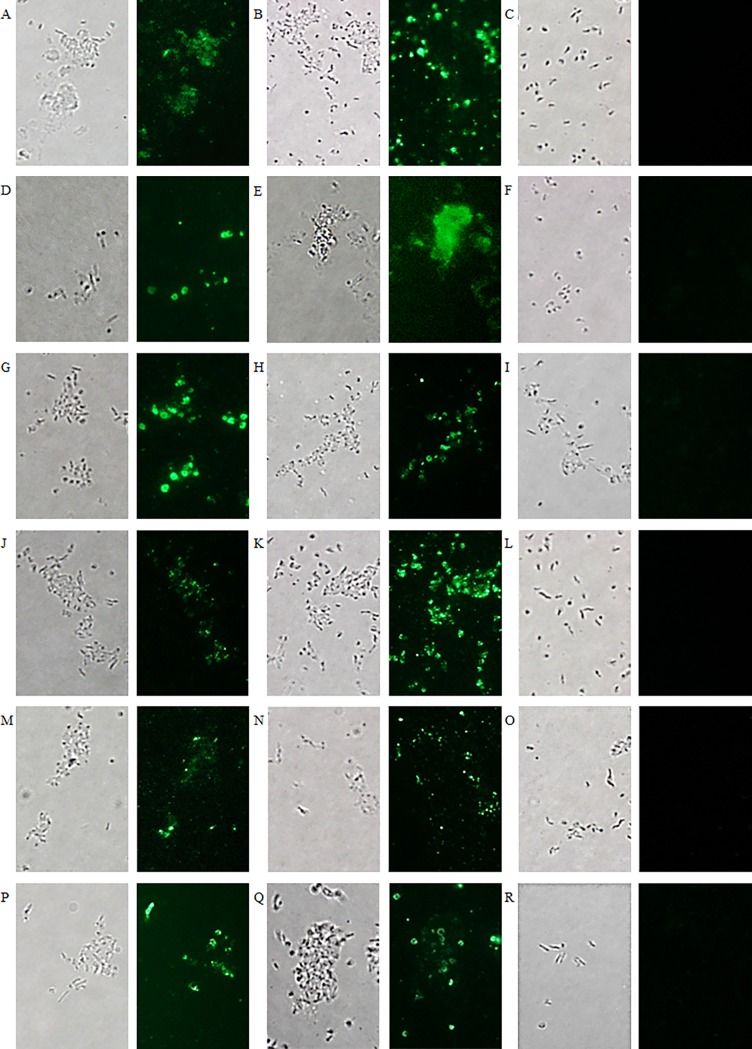
Visualisation of the binding of chimeric anti-MOMP antibodies in seed extract with *Campylobacter* isolates. (A, B, C, J, K, L) *C*. *jejuni* strain KC40, (D, E, F, M, N, O) *C*. *jejuni* strain Cam12/0156 and (G, H, I, P, Q, R) *C*. *coli* strain K43/5. Binding with the different *Campylobacter* isolates is shown with seed extract containing (A, D, G) Nb5-IgA, (B, E, H) Nb23-IgA, (C, F, I) V1-IgA, (J, M, P) Nb5-IgY, (K, N, Q) Nb23-IgY and (L, O, R) V1-IgY. As a negative control, the nanobody V1 against F4-fimbriated *E*. *coli* was used. Bright field microscopy was used for the visualisation of the corresponding bacterial cells.

To verify whether the chimeric antibodies produced in seeds bind their corresponding recombinant antigens and *C*. *jejuni* bacteria, ELISA was performed (Fig 6). The binding between coated purified MOMP or KC40 bacterial cells and twofold serial dilutions of the seed extracts, ranging from undiluted to 1/1024, was measured. Extract of non-transformed *A*. *thaliana* seeds was used as a negative control. The obtained binding curves show interaction at low concentration of the chimeric antibodies with the bacteria as well as with the purified MOMP. The same downward trend was observed in both cases. The binding of the chimeric antibodies with *C*. *jejuni* KC40 was confirmed by immunofluorescence microscopy (Fig 7). Results also indicated the binding of the chimeric antibodies with two additional *Campylobacter* isolates, the *C*. *coli* isolate K43/5 and the human clinical *C*. *jejuni* isolate Cam12/0156. The negative control, the V1 nanobody fused to chicken IgA or IgY, showed no interaction with the selected isolates.

**Fig 8:**
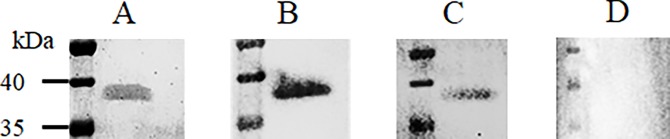
Interaction of chimeric antibodies in seed extract from homozygous plants with their antigen. SDS-PAGE and a western blot were performed on purified MOMP under non-denaturing conditions. (A) SDS-PAGE with purified native MOMP stained with Coomassie blue dye. The results of the western blot confirm the interactions of (B) Nb23-IgA14D and (C) Nb23-IgY12C, with MOMP. (D) Wild-type extract was used as a negative control. The western blot was developed with anti-IgA or anti-IgY antibodies conjugated to HRP.

Comparable expression levels were obtained for the Nb23 chimeric antibodies in the T4 seeds of the obtained homozygous and heterozygous plants in ELISA (S2 Fig). The interaction of the chimeric antibodies in the seed extracts of the homozygous plants with the MOMP protein was confirmed in a western blot (Fig 8). Because nanobodies typically interact with conformational epitopes [37,50–52], a non-denaturing SDS-PAGE was used. Native MOMP corresponds in SDS-PAGE with a protein band with an apparent molecular weight of 38 kDa. A clear band was observed when seed extract was used of plants transformed with the Nb23-IgA and Nb23-IgY constructs, confirming the interaction.

### The anti-flagellin nanobodies bind to *C*. *jejuni* bacterial cells

**Fig 9:**
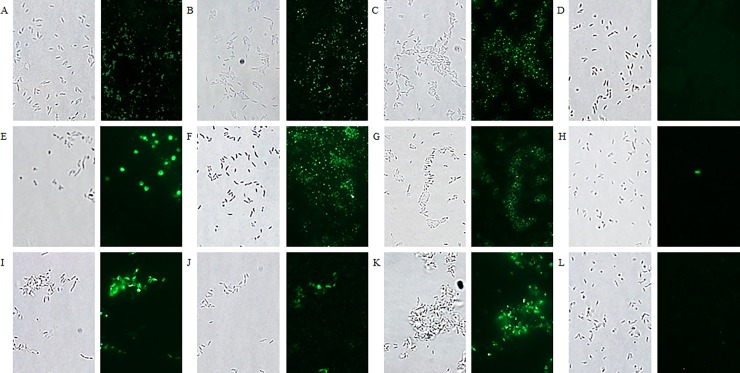
Fluorescence microscopy visualising the binding of labelled anti-flagellin nanobodies with different *Campylobacter* isolates. (A, B, C, D) *C*. *jejuni* strain KC40, (E, F, G, H) *C*. *jejuni* strain Cam12/0156 and (I, J, K, L) *C*. *coli* strain K43/5. Interaction with the different isolates is shown with (A, E, I) Nb2Flag8, (B, F, J) Nb2Flag24 and (C, G, K) Nb2Flag67. (D, H, L) The negative control, fluorescently labelled V1 directed against F4-fimbriated *E*. *coli*, did not bind with the *Campylobacter* bacteria. The corresponding bacterial cells were visualised by bright field microscopy.

Purified flagella (S1 File) were used for the isolation of clones encoding anti-flagellin nanobodies from the nanobody library (S2 File). After panning, three anti-flagellin nanobodies (Nb2Flag8, Nb2Flag24 and Nb2Flag67) (Figure C in S2 File) were selected. ELISA confirmed the interaction of the three nanobodies with purified flagella and motile *C*. *jejuni* KC40 bacteria (S3 Fig). Immunofluorescence microscopy validated this result (Fig 9) and also revealed their binding with the human clinical *C*. *jejuni* isolate Cam12/0156, and the *C*. *coli* isolate K43/5. This indicates that these nanobodies may interact with conserved regions of the flagellins. The V1 nanobody, directed against the FaeG subunit of *E*. *coli* F4 fimbriae, showed no interaction.

### Transient expression of anti-flagellin nanobodies anchored to the Fc region of chicken IgA in leaves

**Fig 10:**
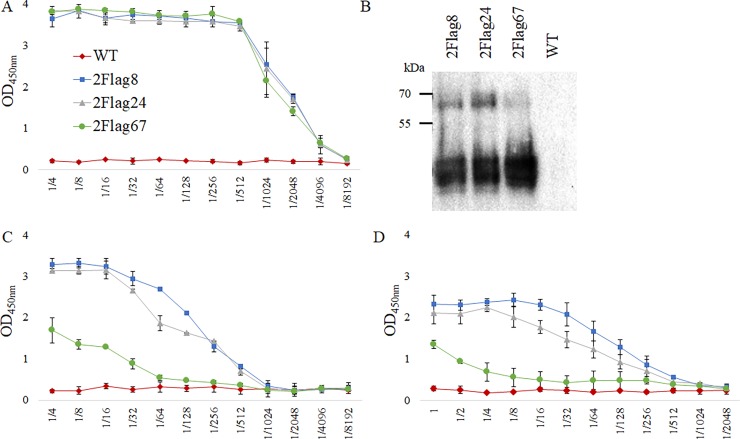
Transient expression of anti-flagellin chimeric antibodies in *N*. *benthamiana* leaves and the interaction with purified flagellins and motile *C*. *jejuni* KC40. (A) ELISA for the detection of chimeric antibodies in serial dilutions (1/4–1/8192) of extracts of infiltrated leaves. (B) Western blot results of leaf extracts with Nb2Flag8, Nb2Flag24 and Nb2Flag67 nanobodies fused to the Fc-domain of chicken IgA. The western blot was developed with anti-IgA conjugated to HRP. As a negative control, leaf extract of wild-type *N*. *benthamiana* was used. (C) Binding curve of twofold serial dilutions (1/4–1/8192) of leaf extract with coated purified flagellins. (D) Binding curve of twofold serial dilutions (undiluted– 1/2048) of leaf extract with coated *C*. *jejuni* KC40 bacteria. Anti-IgA conjugated to HRP was used for the development of the ELISA.

**Fig 11:**
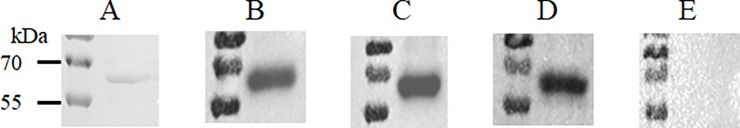
Visualisation of the interaction of chimeric antibodies in extracts of *N*. *benthamiana* leaves with purified flagellins. Non-denatured purified flagellins were used in SDS-PAGE and a western blot. (A) Purified flagellins on SDS-PAGE stained with Coomassie blue dye. Western blotting confirms the interactions of (B) Nb2Flag8-IgA, (C) Nb2Flag24-IgA and (D) Nb2Flag67-IgA. (E) Wild-type extract was used as a negative control. Anti-IgA antibodies conjugated to HRP were used for the development of the western blot.

Nanobodies (Nb2Flag8, Nb2Flag24 and Nb2Flag67) directed against the flagellins of *C*. *jejuni* were fused to the Fc-domain of chicken IgA and cloned in the pEAQ-*HT*-DEST1 vector. The obtained expression vectors (pGV5923, pGV5925 and pGV5927) (Table 1) were transformed into *A*. *tumefaciens* LBA4404, which was subsequently used for infiltration of *N*. *benthamiana* leaves. Extracts of these leaves were analysed for the presence of chimeric antibodies via ELISA and western blotting. ELISA plates were coated with a serial dilution of the leaf extracts (Fig 10A). Development, using anti-IgA conjugated to HRP, clearly confirmed the transient expression of the chimeric antibodies in leaves. The ELISA results indicated no significant difference in expression between the three constructs. The interaction of the chimeric antibodies with purified flagellins and motile *C*. *jejuni* KC40 was confirmed by ELISA (Fig 10C and 10D). To test the interaction with flagellins and bacterial cells, serial dilutions of the extracts were used. In both cases, clear dosage-dependent binding was observed. The results show a lower binding for the extract containing Nb2Flag67-IgA chimeric antibodies, while previous ELISA results showed that the Nb2Flag67 nanobody is not a poorer binder than Nb2Flag8 or Nb2Flag24 (S3 Fig). This can possibly be explained by the more extensive degradation of the Nb2Flag67-IgA construct in the leaf extract, which was observed in the western blot (Fig 10B). The bands of lower molecular weight show extensive proteolytic degradation. The interaction of the chimeric antibodies with native purified flagellins was confirmed in a non-denaturing western blot (Fig 11). The flagellins correspond with the protein band of an apparent molecular weight of 63 kDa.

### Motility of *Campylobacter* is reduced by anti-flagellin chimeric antibodies produced in seeds

**Fig 12:**
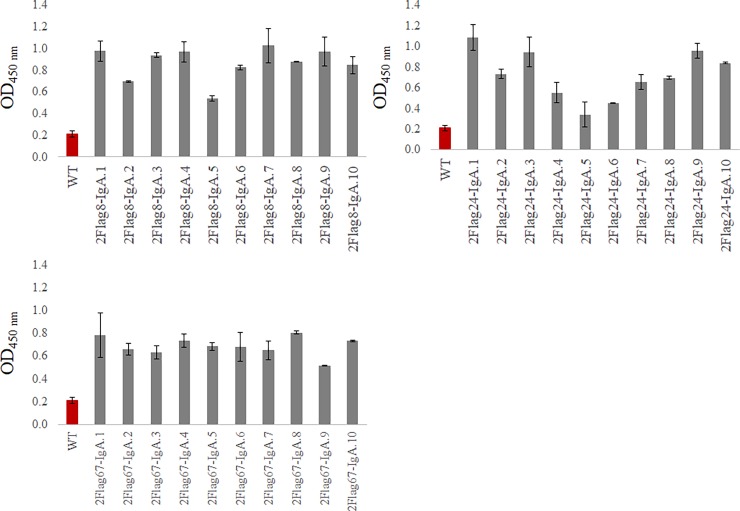
Expression of chimeric antibodies in transgenic *A*. *thaliana* seeds. Extracts of T2 seeds were analysed by ELISA. The presence of chimeric antibodies in the extracts was tested, using anti-chicken IgA conjugated to HRP. An extract of wild-type *A*. *thaliana* seeds was used as a negative control. The error bars represent the standard deviation.

Similarly as for the expression of the anti-MOMP antibodies, the constructs encoding anti-flagellin Nb-IgA antibodies were cloned in the pPhasGW vector. The resulting plasmids (pGV5923, pGV5926 and pGV5928) (Table 1) were transformed into *A*. *tumefaciens* C58C1 Rif^R^ (pMP90). The resulting strains were used for transformation of *A*. *thaliana* Col-0 using the floral dip method [49]. Three plants were transformed with each construct. The T2 seeds of ten kanamycin-resistant plants were analysed for each anti-flagellin chimeric antibody (Fig 12).

**Fig 13:**
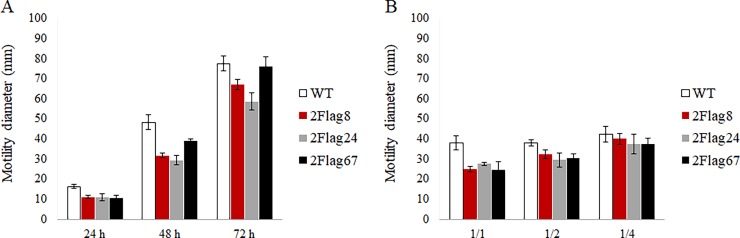
Motility of *C*. *jejuni* KC40 after incubation with chimeric antibodies produced in seeds. The bacterial spread through the soft-agar, represented by the diameter of the circle, was measured. (A) The mean diameter (mm ± SD) was plotted versus the incubation time. (B) The influence of different dilutions (1/1–1/4) of the seed extract containing the chimeric antibodies on the motility of *Campylobacter* after 48 h, was analysed. The mean diameter (mm ± SD) was plotted.

Seed extracts containing the anti-flagellin antibodies were used in a soft-agar assay to assess the influence of the chimeric antibodies on the motility of *Campylobacter* bacteria (Fig 13). *C*. *jejuni* KC40 bacteria were incubated with extract containing Nb2Flag8-IgA.1, Nb2Flag24-IgA.1 and Nb2Flag67-IgA.1. An extract of wild-type *Arabidopsis thaliana* Col-0 seeds was used as a control. After 48 hours, a reduction was observed in the bacterial motility for the three chimeric antibodies (Fig 13A). The influence of different dilutions of the chimeric antibodies on the *Campylobacter* motility after 48 hours of incubation was analysed. Fig 13B shows that the inhibitory effect of the chimeric antibodies diminished when the seed extracts were diluted.

## Discussion

Passive immunisation, using pathogen-specific antibodies, can be successfully applied in human and veterinary medicine. Oral delivery of IgY antibodies, derived from chicken eggs, effectively inhibited colonisation of broilers by different pathogens, such as *Salmonella* and *Campylobacter* [10,53–55]. Specific egg-derived IgY antibodies protected piglets against enterotoxigenic *E*. *coli* [56]. Passive immunisation using IgA derivatives, also protected against enterotoxigenic *E*. *coli* [23,57].

Nanobody-based chimeric antibodies have been developed in this study for a similar passive immunisation strategy to prevent colonisation of chickens by *Campylobacter* or reducing pathogen loads in the intestinal tract. Recombinant bivalent chimeric heavy-chain only antibodies were constructed, via fusion of anti-*Campylobacter* nanobodies with the effector domains of IgA or IgY. Nanobodies against the MOMP and flagella of *Campylobacter* were isolated and shown to interact with different *Campylobacter* strains. The latter can be important, because broilers can be colonised by a large variety of *Campylobacter* strains [58,59]. MOMP is a virulence factor and has an essential transport and structural function [30,60]. The ability of multimers of these anti-MOMP nanobodies to agglutinate *Campylobacter* bacteria, has been demonstrated [27]. Another important virulence factor is the flagellum, required for motility, chemotaxis and transport of non-flagellar proteins [61–63]. Nanobodies directed against *Campylobacter* flagellin, fused to a pentameric protein, were shown to inhibit the motility of *Campylobacter* and the colonisation of chicks [64].

Chimeric antibodies, directed against MOMP or flagella, were produced in plants as additional tools to develop novel strategies for the protection of chicks against *Campylobacter* colonisation. Easy genetic manipulation is possible in plants and the absence of endotoxins and mammalian pathogens are an advantage for biosecurity and for therapeutic use [65,66]. The cost of expression of recombinant proteins in plants, is estimated to be lower compared with other production platforms [67]. Transient expression of the constructs in leaves of *N*. *benthamiana* showed that antibodies recognising their respective antigens were produced at low expression levels. In addition, smaller proteins, possibly generated by proteolytic degradation, were also observed in the western blots with monoclonal anti-His antibody, notwithstanding the use of a cocktail of proteinase inhibitors in the extraction buffer. Proteolytic degradation is also observed in other studies [23,68]. Whether these degraded proteins are present in the plant tissues or are generated by residual proteolytic activity during extraction is unclear.

For passive immunisation of broilers, sufficient amounts of antibodies are required. Therefore, the genes encoding the chimeric antibodies were expressed in seeds of transgenic *Arabidopsis* plants. Seeds are ideal for the expression and storage of recombinant proteins because they are natural storage organs of the plant, containing large quantities of proteins, and show low protease activity during storage [69]. Oral administration of pea seeds expressing a single chain Fv antibody mitigated *Eimeria* infections in chickens [26]. Also, addition of *A*. *thaliana* seeds expressing a chimeric porcine IgA directed against a fimbrial adhesin to the feed was already used successfully to protect piglets against enterotoxigenic *E*. *coli* infection [23]. No adverse effects were reported in these studies. The safety and the performance of genetically modified crops in diets of broiler chickens and laying hens were evaluated in several poultry nutrition studies. These studies showed that transgenic crops provided comparable performance, carcass and egg yields, and meat and egg composition, when compared with conventional grains (reviewed in Tufarelli *et al*., 2015) [70]. Other studies also evaluated whether foreign DNA and proteins could be detected in meat, egg, and tissue samples from broiler chickens and laying hens fed diets containing transgenic crops. None of these studies could detect transgenic DNA or proteins in food products derived from these animals, using the most sensitive detection methods available (reviewed in Tufarelli *et al*., 2015) [70].

The results of this study show that the chimeric antibodies were produced in comparable quantities in seeds of heterozygous as well as homozygous plants. With a few exceptions, the variation of the expression levels was relatively low and little degradation products were observed in western blots. The accumulation level of the IgA chimeric antibodies in *Arabidopsis* seeds is around 1% of total soluble protein (TSP), and for the IgY chimeric antibodies yields of up to 8% TSP were obtained. These results are comparable with those observed previously [71,72]. ELISA and western blot confirmed that the chimeric antibodies in seed extracts recognise the corresponding purified antigen and also bind intact *Campylobacter* bacteria. The motility assay showed a significant reduction of the motility *of C*. *jejuni* KC40 in the presence of anti-flagellin chimeric antibodies produced *in planta*. Higher concentrations of the chimeric antibodies or the addition of the antibodies to the soft agar medium may lead to an higher reduction.

In conclusion, functional chimeric antibodies, recognising flagella and MOMP, were successfully produced in *N*. *benthamiana* leaves and *A*. *thaliana* seeds. The homozygous transgenic lines were upscaled to obtain sufficient quantities of transgenic seeds to test the *in vivo* effect of passive immunisation on colonisation of chickens by *Campylobacter*. If the results are positive, the chimeric antibodies can be expressed in seeds of crop plants, to produce amounts of antibodies needed in field conditions.

## Supporting information

S1 File Purification of flagellin of the *C*. *jejuni* strain KC40 flagella. (PDF)

S2 File Phage library construction and selection of anti‑flagellin *Campylobacter* nanobodies. (PDF)

S3 File Thermal and pH stability of nanobodies. (PDF)

S4 File Construction of the synthetic Nb-IgY or Nb-IgA fusion genes. (PDF)

S1 Fig Immunofluorescence microscopy confirms the interaction of Nb5 and Nb23 with *Campylobacter* isolates. (A, B, C) *C*. *jejuni* strain KC40, (D, E, F) *C*. *jejuni* strain Cam12/0156 and (G, H, I) *C*. *coli* strain K43/5. The binding of Nb5 is shown in A, D and G and the binding of Nb23 in B, E and H. As a negative control, the fluorescently labelled (C, F, I) V1 nanobody was used.
(TIF)

S2 Fig Expression of chimeric antibodies in seeds of homozygous and heterozygous plants. ELISA was used for the analysis of seed extracts from *A*. *thaliana* plants transformed with (A) Nb23-IgA constructs and (B) Nb23-IgY constructs. The results of the extracts of the homozygous plants are visualised by the histogram with hatched shading. Extract of wild-type *A*. *thaliana* seeds was used as a negative control. The ELISA was developed using anti-IgA or anti-IgY conjugated to HRP. The error bars correspond to the standard deviation.
(TIF)

S3 Fig ELISA for the confirmation of the interaction of anti-flagellin nanobodies with (A) purified flagellins and (B) *C*. *jejuni* KC40. Bound His-tagged nanobodies were detected with mouse anti-histidine monoclonal antibodies and goat anti-mouse IgG. The error bars correspond to the standard deviation.
(TIF)

S1 Table Determination of the chimeric antibody concentration in extracts of *A*. *thaliana* seeds with the Chicken IgA ELISA Kit. (PDF)

## References

[1] Kaakoush NO, Castaño-Rodríguez N, Mitchell HM, Man SM. Global epidemiology of Campylobacter infection. Clin Microbiol Rev. 2015;28(3):687–720. 10.1128/CMR.00006-15 26062576 PMC4462680

[2] EFSA. The European Union summary report on trends and sources of zoonoses, zoonotic agents and food-borne outbreaks in 2014 European Food Safety Authority European Centre for Disease Prevention and Control. EFSA J. 2014;13(12):4329.

[3] Newell DG, Fearnley C. Sources of Campylobacter colonization in broiler chickens. Appl Enviromental Microbiol. 2003;69(8):4343–51. 10.1128/AEM.69.8.4343-4351.2003 PMC169125 12902214

[4] Blaser M. Epidemiologic and clinical features of Campylobacter jejuni infections. J Infect Dis. 1997;176(Suppl 2):S103–5. 9396691 10.1086/513780

[5] Moore JE, Corcoran D, Dooley JSG, Fanning S, Lucey B, Matsuda M, et al Campylobacter. Vet Res. 2005;36(3):351–82. 10.1051/vetres:2005012 15845230

[6] Näther G, Alter T, Martin A, Ellerbroek L. Analysis of risk factors for Campylobacter species infection in broiler flocks. Poult Sci. 2009;88(6):1299–305. 10.3382/ps.2008-00389 19439643

[7] Sahin O, Luo N, Huang S, Zhang Q. Effect of Campylobacter-specific maternal antibodies on Campylobacter jejuni colonization in young chickens. Appl Enviromental Microbiol. 2003;69(9):5372–9. 10.1128/AEM.69.9.5372-5379.2003 PMC194908 12957925

[8] Lee MD, Newell DG. Campylobacter in poultry: filling an ecological niche. Avian Dis. 2006 3;50(1):1–9. 10.1637/7474-111605R.1 16617973

[9] Lin J. Novel approaches for Campylobacter control in poultry. Foodborne Pathog Dis. 2009 9;6(7):755–65. 10.1089/fpd.2008.0247 19425824 PMC3145176

[10] Hermans D, Van Steendam K, Verbrugghe E, Verlinden M, Martel A, Seliwiorstow T, et al Passive immunization to reduce Campylobacter jejuni colonization and transmission in broiler chickens. Vet Res. 2014;45:27 10.1186/1297-9716-45-27 24589217 PMC3996517

[11] Messens W, Hartnett E, Gellynck X, Viaene J, Halet D, Herman L, et al Quantitative risk assessment of human campylobacteriosis through the consumption of chicken meat in Belgium. 18th Eur Symp Qual Poult Meat; 12th Eur Symp Qual Eggs Egg Prod. 2007;167–8.

[12] Hermans D, Van Deun K, Messens W, Martel A, Van Immerseel F, Haesebrouck F, et al Campylobacter control in poultry by current intervention measures ineffective: urgent need for intensified fundamental research. Vet Microbiol. 2011 9;152(3–4):219–28. 10.1016/j.vetmic.2011.03.010 21482043

[13] Moore JE, Barton MD, Blair IS, Corcoran D, Dooley JSG, Fanning S, et al The epidemiology of antibiotic resistance in Campylobacter. Microbes Infect. 2006;8(7):1955–66. 10.1016/j.micinf.2005.12.030 16716632

[14] de Zoete MR, van Putten JPM, Wagenaar JA. Vaccination of chickens against Campylobacter. Vaccine. 2007;25(30):5548–57. 10.1016/j.vaccine.2006.12.002 17224215

[15] Al-Adwani SR, Crespo R, Shah DH. Production and Evaluation of Chicken Egg-Yolk-Derived Antibodies Against Campylobacter jejuni Colonization-Associated Proteins. Foodborne Pathog Dis. 2013;10(7):624–31. 10.1089/fpd.2012.1313 23742296

[16] Muyldermans S, Baral TN, Retamozzo VC, De Baetselier P, De Genst E, Kinne J, et al Camelid immunoglobulins and nanobody technology. Vet Immunol Immunopathol. 2009;128(1–3):178–83. 10.1016/j.vetimm.2008.10.299 19026455

[17] Harmsen MM, De Haard HJ. Properties, production, and applications of camelid single-domain antibody fragments. Appl Microbiol Biotechnol. 2007;77(1):13–22. 10.1007/s00253-007-1142-2 17704915 PMC2039825

[18] Roche AM, Richard AL, Rahkola JT, Janoff EN, Weiser JN. Antibody blocks acquisition of bacterial colonization through agglutination. Mucosal Immunol. 2015;8(1):176–85. 10.1038/mi.2014.55 24962092 PMC4268183

[19] Kontermann RE. Strategies to extend plasma half-lives of recombinant antibodies. BioDrugs. 2009;23(2):93–109. 10.2165/00063030-200923020-00003 19489651

[20] Hutt M, Färber-Schwarz A, Unverdorben F, Richter F, Kontermann RE. Plasma half-life extension of small recombinant antibodies by fusion to immunoglobulin-binding domains. J Biol Chem. 2012;287(7):4462–9. 10.1074/jbc.M111.311522 22147690 PMC3281650

[21] Kontermann RE. Strategies for extended serum half-life of protein therapeutics. Current Opinion in Biotechnology. 2011 p. 22:868–876. 10.1016/j.copbio.2011.06.012 21862310

[22] Schirrmann T, Al-halabi L, Dübel S, Hust M. Production systems for recombinant antibodies. Front Biosci. 2008;13(13):4576–94. 18508530 10.2741/3024

[23] Virdi V, Coddens A, De Buck S, Millet S, Goddeeris BM, Cox E, et al Orally fed seeds producing designer IgAs protect weaned piglets against enterotoxigenic Escherichia coli infection. Proc Natl Acad Sci U S A. 2013;110(29):11809–14. 10.1073/pnas.1301975110 23801763 PMC3718133

[24] Yusibov V, Kushnir N, Streatfield SJ. Antibody production in plants and green algae. Annu Rev Plant Biol. 2016;67(1):669–701. 26905655 10.1146/annurev-arplant-043015-111812

[25] Rademacher T, Arcalis E, Stoger E. Production and localization of recombinant pharmaceuticals in transgenic seeds. Faye L, Gomord V, editors. Methods Mol Biol. 2009;483:69–87. 10.1007/978-1-59745-407-0_5 19183894

[26] Zimmermann J, Saalbach I, Jahn D, Giersberg M, Haehnel S, Wedel J, et al Antibody expressing pea seeds as fodder for prevention of gastrointestinal parasitic infections in chickens. BMC Biotechnol. 2009;9:79 10.1186/1472-6750-9-79 19747368 PMC2755478

[27] Vanmarsenille C, Díaz del Olmo I, Elseviers J, Hassanzadeh Ghassabeh G, Moonens K, Vertommen D, et al Nanobodies targeting conserved epitopes on the major outer membrane protein of Campylobacter as potential tools for control of Campylobacter colonization. Vet Res. 2017;48(1):86 10.1186/s13567-017-0491-9 29216932 PMC5721652

[28] Guerry P. Campylobacter flagella: not just for motility. Trends Microbiol. 2007;15(10):456–61. 10.1016/j.tim.2007.09.006 17920274

[29] Moser I, Schroeder W, Salnikow J. Campylobacter jejuni major outer membrane protein and a 59-kDa protein are involved in binding to fibronectin and INT 407 cell membranes. FEMS Microbiol Lett. 1997;157(2):233–8. 9435102 10.1111/j.1574-6968.1997.tb12778.x

[30] Flanagan RC, Neal-McKinney JM, Dhillon AS, Miller WG, Konkel ME. Examination of Campylobacter jejuni putative adhesins leads to the identification of a new protein, designated FlpA, required for chicken colonization. Infect Immun. 2009;77(6):2399–407. 10.1128/IAI.01266-08 19349427 PMC2687328

[31] Lebacq-Verheyden A-M, Vaerman J-P, Heremans JF. Quantification and distribution of chicken immunoglobulins IgA, IgM and IgG in serum and secretions. Immunology. 1974;27(4):683–92. 4215742 PMC1445738

[32] Carlander D, Stålberg J, Larsson A. Chicken antibodies. Ups J Med Sci. 1999;104(3):179–90. 10680951 10.3109/03009739909178961

[33] Van Deun K, Pasmans F, Ducatelle R, Flahou B, Vissenberg K, Martel A, et al Colonization strategy of Campylobacter jejuni results in persistent infection of the chicken gut. Vet Microbiol. 2008;130(3–4):285–97. 10.1016/j.vetmic.2007.11.027 18187272

[34] Hobb RI, Fields JA, Burns CM, Thompson SA. Evaluation of procedures for outer membrane isolation from Campylobacter jejuni. Microbiology. 2009 3;155(Pt 3):979–88. 10.1099/mic.0.024539-0 19246768 PMC2763183

[35] Bolla JM, Loret E, Zalewski M, Pages JM. Conformational analysis of the Campylobacter jejuni porin. J Bacteriol. 1995;177(15):4266–71. 7543469 10.1128/jb.177.15.4266-4271.1995 PMC177172

[36] Logan SM, Trust TJ. Molecular identification of surface protein antigens of Campylobacter jejuni. Infect Immun. 1983;42(2):675–82. 6642648 10.1128/iai.42.2.675-682.1983 PMC264482

[37] Pardon E, Laeremans T, Triest S, Rasmussen SGF, Wohlkönig A, Ruf A, et al A general protocol for the generation of nanobodies for structural biology. Nat Protoc. 2014 3;9(3):674–93. 10.1038/nprot.2014.039 24577359 PMC4297639

[38] Conrath KE, Lauwereys M, Galleni M, Matagne A, Frère J-M, Kinne J, et al β-Lactamase inhibitors derived from single-domain antibody fragments elicited in the Camelidae. Antimicrob Agents Chemother. 2001 10;45(10):2807–12. 10.1128/AAC.45.10.2807-2812.2001 11557473 PMC90735

[39] Dagert M, Ehrlich SD. Prolonged incubation in calcium chloride improves the competence of Escherichia coli cells. Gene. 1979;6(1):23–38. 383576 10.1016/0378-1119(79)90082-9

[40] Huynh K, Partch CL. Analysis of protein stability and ligand interactions by thermal shift assay. Curr Protoc protein Sci. 2015;79:28.9.1–28.9.14. 25640896 10.1002/0471140864.ps2809s79 PMC4332540

[41] Mansikka A. Chicken IgA H chains. Implications concerning the evolution of H chain genes. J Immunol. 1992;149(3):855–61. 1634774

[42] Parvari R, Avivi A, Lentner F, Ziv E, Tel-Or S, Burstein Y, et al Chicken immunoglobulin gamma-heavy chains: limited VH gene repertoire, combinatorial diversification by D gene segments and evolution of the heavy chain locus. EMBO J. 1988;7(3):739–44. 3135182 10.1002/j.1460-2075.1988.tb02870.x PMC454383

[43] Sainsbury F, Thuenemann EC, Lomonossoff GP. pEAQ: versatile expression vectors for easy and quick transient expression of heterologous proteins in plants. Plant Biotechnol J. 2009;7(7):682–93. 10.1111/j.1467-7652.2009.00434.x 19627561

[44] Hoekema A, Hirsch PR, Hooykaas PJJ, Schilperoort RA. A binary plant vector strategy based on separation of vir- and T-region of the Agrobacterium tumefaciens Ti-plasmid. Nature. 1983;303:179–80.

[45] McCormac AC, Elliott MC, Chen DF. A simple method for the production of highly competent cells of Agrobacterium for transformation via electroporation. Mol Biotechnol. 1998;9(2):155–9. 10.1007/BF02760816 9658392

[46] De Buck S, Virdi V, De Meyer T, De Wilde K, Piron R, Nolf J, et al Production of camel-like antibodies in plants. Methods Mol Biol. 2012;911:305–24. 10.1007/978-1-61779-968-6_19 22886260

[47] Morandini F, Avesani L, Bortesi L, Van Droogenbroeck B, De Wilde K, Arcalis E, et al Non-food/feed seeds as biofactories for the high-yield production of recombinant pharmaceuticals. Plant Biotechnol J. 2011;9(8):911–21. 10.1111/j.1467-7652.2011.00605.x 21481135

[48] Koncz C, Schell J. The promoter of TL-DNA gene 5 controls the tissue-specific expression of chimaeric genes carried by a novel type of Agrobacterium binary vector. Mol Gen Genet. 1986;204(3):383–96.

[49] Clough SJ, Bent AF. Floral dip: a simplified method for Agrobacterium-mediated transformation of Arabidopsis thaliana. Plant J. 1998;16(6):735–43. 10069079 10.1046/j.1365-313x.1998.00343.x

[50] Rudolph MJ, Vance DJ, Cheung J, Franklin MC, Burshteyn F, Cassidy MS, et al Crystal structures of ricin toxin’s enzymatic subunit (RTA) in complex with neutralizing and non-neutralizing single-chain antibodies. J Mol Biol. 2014;426(17):3057–68. 10.1016/j.jmb.2014.05.026 24907552 PMC4128236

[51] Hassanzadeh-Ghassabeh G, Devoogdt N, De Pauw P, Vincke C, Muyldermans S. Nanobodies and their potential applications. Nanomedicine. 2013;8(6):1013–26. 10.2217/nnm.13.86 23730699

[52] Braun MB, Traenkle B, Koch PA, Emele F, Weiss F, Poetz O, et al Peptides in headlock–a novel high-affinity and versatile peptide-binding nanobody for proteomics and microscopy. Sci Rep. 2016;6:19211 10.1038/srep19211 26791954 PMC4726124

[53] Rahimi S, Shiraz ZM, Salehi TZ, Torshizi MAK, Grimes JL. Prevention of Salmonella infection in poultry by specific egg-derived antibody. Int J Poult Sci. 2007;6(4):230–5.

[54] Kovacs-Nolan J, Mine Y. Egg yolk antibodies for passive immunity. Annu Rev Food Sci Technol. 2012;3:163–82. 10.1146/annurev-food-022811-101137 22136128

[55] Baxter D. Active and passive immunity, vaccine types, excipients and licensing. Occup Med (Chic Ill). 2007;57(8):552–6. 10.1093/occmed/kqm110 18045976

[56] Yokoyama H, Peralta RC, Diaz R, Sendo S, Ikemori Y, Kodama Y. Passive protective effect of chicken egg yolk immunoglobulins against experimental enterotoxigenic Escherichia coli infection in neonatal piglets. Infect Immun. 1992;60(3):998–1007. 1347289 10.1128/iai.60.3.998-1007.1992 PMC257586

[57] Corthésy B. Recombinant immunoglobulin A: powerful tools for fundamental and applied research. Trends Biotechnol. 2002;20(2):65–71. 11814596 10.1016/s0167-7799(01)01874-1

[58] Höök H, Fattah MA, Ericsson H, Vågsholm I, Danielsson-Tham ML. Genotype dynamics of Campylobacter jejuni in a broiler flock. Vet Microbiol. 2005;106(1–2):109–17. 10.1016/j.vetmic.2004.12.017 15737480

[59] EFSA. Scientific report of EFSA: analysis of the baseline survey on the prevalence of Campylobacter in broiler batches and of Campylobacter and Salmonella on broiler carcasses in the EU, 2008. EFSA J. 2010;8(03):1503. 10.2903/j.efsa.2011.2017 PMC13090778 42006083

[60] Wu Z, Periaswamy B, Sahin O, Yaeger M, Plummer P, Zhai W, et al Point mutations in the major outer membrane protein drive hypervirulence of a rapidly expanding clone of Campylobacter jejuni. Proc Natl Acad Sci U S A. 2016;113(38):10690–5. 10.1073/pnas.1605869113 27601641 PMC5035848

[61] Gilbreath JJ, Cody WL, Merrell DS, Hendrixson DR. Change is good: variations in common biological mechanisms in the epsilonproteobacterial genera Campylobacter and Helicobacter. Microbiol Mol Biol Rev. 2011;75(1):84–132. 10.1128/MMBR.00035-10 21372321 PMC3063351

[62] Nachamkin I, Yang XH, Stern NJ. Role of Campylobacter jejuni flagella as colonization factors for three-day-old chicks: analysis with flagellar mutants. Appl Environ Microbiol. 1993;59(5):1269–73. 8517729 10.1128/aem.59.5.1269-1273.1993 PMC182076

[63] Konkel ME, Klena JD, Rivera-Amill V, Monteville MR, Biswas D, Raphael B, et al Secretion of virulence proteins from Campylobacter jejuni is dependent on a functional flagellar export apparatus. J Bacteriol. 2004;186(11):3296–303. 10.1128/JB.186.11.3296-3303.2004 15150214 PMC415756

[64] Riazi A, Strong PCR, Coleman R, Chen W, Hirama T, van Faassen H, et al Pentavalent single-domain antibodies reduce Campylobacter jejuni motility and colonization in chickens. Mantis NJ, editor. PLoS One. 2013;8(12):e83928 10.1371/journal.pone.0083928 24391847 PMC3877120

[65] Pogue GP, Vojdani F, Palmer KE, Hiatt E, Hume S, Phelps J, et al Production of pharmaceutical-grade recombinant aprotinin and a monoclonal antibody product using plant-based transient expression systems. Plant Biotechnol J. 2010;8(5):638–54. 20514694 10.1111/j.1467-7652.2009.00495.x

[66] Porro D, Sauer M, Branduardi P, Mattanovich D. Recombinant protein production in yeasts. Mol Biotechnol. 2005;31(3):245–60. 10.1385/MB:31:3:245 16230775

[67] Chen M, Liu X, Wang Z, Song J, Qi Q, Wang PG. Modification of plant N-glycans processing: the future of producing therapeutic protein by transgenic plants. Med Res Rev. 2005;25(3):343–60. 10.1002/med.20022 15499575

[68] De Wilde K, De Buck S, Vanneste K, Depicker A. Recombinant antibody production in Arabidopsis seeds triggers an unfolded protein response. Plant Physiol. 2013;161(2):1021–33. 10.1104/pp.112.209718 23188806 PMC3561000

[69] Boothe J, Nykiforuk C, Shen Y, Zaplachinski S, Szarka S, Kuhlman P, et al Seed-based expression systems for plant molecular farming. Plant Biotechnol J. 2010;8(5):588–606. 10.1111/j.1467-7652.2010.00511.x 20500681

[70] Tufarelli V, Selvaggi M, Dario C, Laudadio V. Genetically Modified Feeds in Poultry Diet: Safety, Performance, and Product Quality. Crit Rev Food Sci Nutr. 2015; 10.1080/10408398.2012.667017 24915369

[71] De Jaeger G, Scheffer S, Jacobs A, Zambre M, Zobell O, Goossens A, et al Boosting heterologous protein production in transgenic dicotyledonous seeds using Phaseolus vulgaris regulatory sequences. Nat Biotechnol. 2002;20(12):1265–8. 10.1038/nbt755 12415287

[72] De Buck S, Nolf J, De Meyer T, Virdi V, De Wilde K, Van Lerberge E, et al Fusion of an Fc chain to a VHH boosts the accumulation levels in Arabidopsis seeds. Plant Biotechnol J. 2013;11(8):1006–16. 10.1111/pbi.12094 23915060

