# Supplementary material for: In planta expression of nanobody-based designer chicken antibodies targeting Campylobacter
Source: PLoS One. 2018 Sep 27;13(9):e0204222. doi: 10.1371/journal.pone.0204222 (PMC6160005; doi:10.1371/journal.pone.0204222)
Supplement: S1 File — (PDF) [file pone.0204222.s001.pdf]

S1 File: Purification of flagellin of the *C. jejuni* strain KC40 flagella

Motile *C. jejuni* produce a flagellum at one or both poles of the cell. The filament of this flagellum is composed of two flagellins, the major flagellin FlaA and the minor flagellin FlaB (Nuijten *et al.*, 1990). The genes encoding FlaA and FlaB are located next to each other (Guerry, 1997). We purified the flagellins FlaA and FlaB of the *C. jejuni* strain KC40 and used these to isolate anti-flagellin nanobodies from an alpaca immunized with heat-inactivated *C. jejuni* KC40 cells (Vanmarsenille *et al.*, 2017).

Purification of the FlaA and FlaB flagellins from *C. jejuni* strain KC40

The *C. jejuni* strain KC40 was grown on 30 9-cm Petri dishes containing Nutrient Broth No.2 agar medium for 48 hours at 42°C under microaerobic conditions (Oxoid CampyGen, Thermo Fisher Scientific). The cells were recovered from the medium by washing with 3 ml sterile H_2O. The flagella were detached in a Warring Model 7011 blender set at the highest speed for two intervals of 30 seconds. The blended suspension was centrifuged at 10000 g for 1 hour at 4°C. The supernatant was saved and the pellet was washed with 30 ml of H_2O. After centrifugation at 10000 g for 1 hour at 4°C, the supernatant was pooled with the first supernatant. The pooled supernatants were centrifuged at 100000 g for 1 hour at 4°C. The pellet containing the flagella was resuspended in 10 ml of H_2O. The previous low- and high-speed centrifugation steps were repeated once. After resuspending the flagella in 10 ml of H_2O, the pH of the suspension was lowered with HCl till 2 and the suspension was incubated on ice for 15 minutes. This solution was centrifuged at 100000 g for 1 hour at 0°C. The pH of the supernatant was then adjusted to 8 with NaOH and the supernatant was incubated for 30 minutes on ice. The protein concentration was determined using a BCA assay (Pierce BCA Protein Assay Kit, Thermo Scientific). The supernatant was aliquoted and kept at -20°C. Samples of the supernatant were analysed by SDS-PAGE and by sequencing using liquid chromatography–tandem mass spectrometry (LC–MS/MS) after trypsin digest. The peptides identified by LC–MS/MS are indicated on the aligned amino acid sequences of FlaA and FlaB (Figure A in S1File).

This analysis showed that the purified flagellin is a mixture of FlaA and FlaB subunits.

```
                                                                 SQANT
                                                                 SQANT
              INTNVAALNAK                      INSAADDASGMAIADSLR
FlaA    1 MGFRINTNVAALNAKANSDLNAKSLDSSLARLSSGLRINSAADDASGMAIADSLRSQANT
          |||||||| ||||| |||  ||  || || |||||||||||||||||||||||||||| |
FlaB    1 MGFRINTNIGALNAHANSVVNANELDKSLSRLSSGLRINSAADDASGMAIADSLRSQAAT
                                               INSAADDASGMAIADSLR
                                                                 SQAAT
                                                                 SQAAT

           LGQAISNGNDALGILQTADKAMDEQLK
           LGQAISNGNDALGILQTADK                    QDGQSLK           LM
FlaA    61 LGQAISNGNDALGILQTADKAMDEQLKILDTIKTKATQAAQDGQSLKTRTMLQADINKLM
           ||||| ||||| ||||||||||||||||||||||||||||||||||||||||||||| ||
FlaB    61 LGQAINNGNDAIGILQTADKAMDEQLKILDTIKTKATQAAQDGQSLKTRTMLQADINRLM
           LGQAINNGNDAIGILQTADK                    QDGQSLK  TMLQADINR
           LGQAINNGNDAIGILQTADKAMDEQLK

           EELDNIANTTSFNGK
FlaA   121 EELDNIANTTSFNGKQLLSGGFTNQEFQIGSSSNQTVKATIGATQSSKIGVTRFETGSQS
           |||||||||| ||||||||| |||||||||   |||||| || || ||||||||||||||
FlaB   121 EELDNIANTTAFNGKQLLSGNFTNQEFQIGSSSNQTIKASIGPTQSSKIGVTRFETGSQS

FlaA   181 VSSGVVGLTIKNYNGIEDFKFDNVVISTSVGTGLGALAEEINRNADKTGVRATYDVKTVG
             |||||||||||||||||||| |||||||||||||||||||   ||||||||||| | |
FlaB   181 FTSGVVGLTIKNYNGLEDFKFDSVVISTSVGTGLGALAEEINKSSDKTGVRATYDVRTTG

FlaA   241 AYAIKEGTTSQDFAINGVVIGKVDYKDGDGNGSLISAINAVKDTTGVQASKDESGKLVLT
            |||||| ||||||||||||||||||||| || |||||| ||||||||||||| ||||||
FlaB   241 VYAIKEGSTSQDFAINGVVIGQINYKDGDNNGQLISAINSVKDTTGVQASKDENGKLVLT

FlaA   301 SADGRGIKITGSIGPGAGIL--QTENYGRLSLVKNDGRDINIGGTNLSAIGMGAADMISQ
           ||||||||||| || | |||  | |||||||||||||||||| ||||| ||||  |||||
FlaB   301 SADGRGIKITGDIGVGSGILSNQKENYGRLSLVKNDGRDINISGTNLSTIGMGTTDMISQ

FlaA   359 ASVSLRESKGQISAANADAMGFNSYNGGGAKQIVIA-SSIGAFMSQAGSGFSKGSGFSVG
           |||||||||||||| |||||||||| |||      | ||| ||||  |||||||||||||
FlaB   361 ASVSLRESKGQISATNADAMGFNSYKGGGKFVFTQAVSSISAFMSASGSGFSKGSGFSVG

FlaA   418 SGKNYSTVLSGSVQIVSSAASISNTYVVSKGSGFSSGSGNSQFAALKTSTVSAHEATAGV
           |||| |  |     ||||||| ||||||| |||||||||||||||||         ||||
FlaB   421 SGKNLSVGLTEGIKIVSSAASMSNTYVVSSGSGFSSGSGNSQFAALKATAANTTDETAGV

               GAMAVMDIAETAITNLDQIR                                DVDF
FlaA   478 TTLKGAMAVMDIAETAITNLDQIRADIGSIQNQVTSTINNITVTQVNVKSAESQIRDVDF
           ||||||||||||||||||||||||||||| |||   ||||||||||||| ||| ||||||
FlaB   481 TTLKGAMAVMDIAETAITNLDQIRADIGSVQNQLQVTINNITVTQVNVKAAESTIRDVDF
                                   ADIGSVQNQLQVTINNITVTQVNVK
               GAMAVMDIAETAITNLDQIR

           ASESANYSK
FlaA   538 ASESANYSKANILAQSGSYAMAQANSSQQNVLRLLQSA*
           |||||| || ||||||||||| |||  ||||| |||||
FlaB   541 ASESANFSKYNILAQSGSYAMSQANAVQQNVLKLLQSA*
                    YNILAQSGSYAMSQANAVQQNVLK
```

Figure A in S1 File. Results of the LC–MS/MS analysis of trypsin-digested purified FlaA and FlaB flagellins of *C. jejuni*. The amino acid sequence of the FlaA and FlaB flagellins of *C. jejuni* strain KC40 are aligned. The peptides obtained after LC–MS/MS analysis are highlighted in yellow (FlaA-specific peptides) or blue (FlaB-specific peptides).

References

Guerry, P. (1997). Non-lipopolysaccharide surface antigens of *Campylobacter* species. *J. Infect. Dis.* 176: 122-124.

Nuijten, P.J.M., Van Asten, F.J.A.M., Gaastra, W. & Van der Zeijst, B.A.M. (1990). Structural and functional analysis of two *Campylobacter jejuni* flagellin genes. *J. Biol. Chem.* 265: 17798-17804.

Vanmarsenille, C., Díaz del Olmo, I., Elseviers, J., Ghassabeh, G.H., Moonens, K., Vertommen, D., *et al.* (2017). Nanobodies targeting conserved epitopes on the Major Outer Membrane Protein of *Campylobacter* as potential tools for control of *Campylobacter* colonization. *Vet Res.* 48: 86.
