# Supplementary material for: In planta expression of nanobody-based designer chicken antibodies targeting Campylobacter
Source: PLoS One. 2018 Sep 27;13(9):e0204222. doi: 10.1371/journal.pone.0204222 (PMC6160005; doi:10.1371/journal.pone.0204222)
Supplement: S2 File — (PDF) [file pone.0204222.s002.pdf]

S2 File: Phage library construction and selection of anti-flagellin *Campylobacter* nanobodies

The detailed protocol for immunization, generation of a nanobody library and selection by phage panning as described by Pardon *et al.* (2014) was followed. *C. jejuni* KC40 cells were heat-inactivated at 55°C for 1 hour. An emulsion (2 ml) was prepared consisting of 1 ml of 1.6×10^8 heat-inactivated *C. jejuni* KC40 cells mixed with an equal volume of the GERBU adjuvant. An alpaca was injected subcutaneously six times at weekly intervals with 2 ml of this emulsion. After week 7, peripheral lymphocytes were isolated from the blood of the immunized alpaca, from which RNA was isolated and converted to cDNA (Pardon *et al.*, 2014) using primers CALL001 (5'-GTCCTGGCTG CTCTTCTACAAGG-3') and CALL002 (5'-GGTACGTGCTGTTGAACTGTTCC-3'). The cDNA was used as template to amplify the sequences encoding the variable domains of heavy chain antibodies using the primers VHH-Back (5'-GATGTGCAGCTGCAGGATCTGGRGGAGG-3' containing a PstI restriction site) and VHH-For (5'-CTAGTGCGGCCGCTGAGACGGTGACCTGGGT-3' containing a BstEII restriction site). The resulting PCR fragments were cloned in the PstI-BstEII digested phage display vector pHEN4 (Arbabi Ghahroudi *et al.*, 1997) and transformed in *E. coli* TG1 cells.

Phage display was used for the isolation of *Campylobacter*-specific nanobodies from this immune library. The phage library was panned twice against the purified flagellins (0.1 µg per well) and uncoated wells. Serial tenfold dilution of binding phages (from undiluted up to 10^{-5}) from the second panning were eluted and used to infect of *E. coli* TG1 (Figure A in S2 File). As can be seen from this figure, 100-fold more colonies were obtained after infection with phages eluted from the flagellin-coated well than from the uncoated well (negative control).

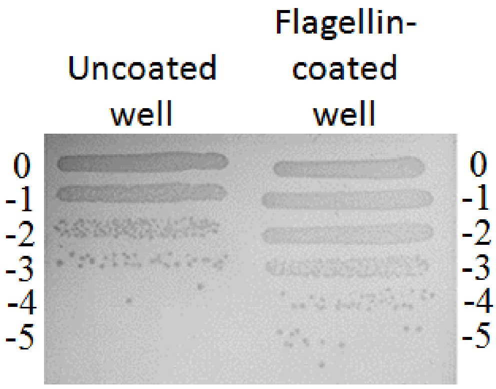

Figure A in S2 File. Plating of *E. coli* TG1 culture infected with tenfold serial dilutions (from undiluted up to 10^{-5}) of eluted phages from the second panning. The TG1 cultures were streaked on LB agar medium supplemented with ampicillin (100 µg/ml) and glucose (2%).

A periplasmic extract was prepared from 96 individual *E. coli* TG1 transformants and used in an ELISA. To confirm their specificity for the flagellins of *Campylobacter,* the periplasmic extracts were incubated with a flagellin-coated (0.1 µg per well; positive) and a non-coated (negative) well. The ratio between the flagellin-coated (positive) well and the negative well was determined after one hour of incubation and is shown in Figure B in S2 File.

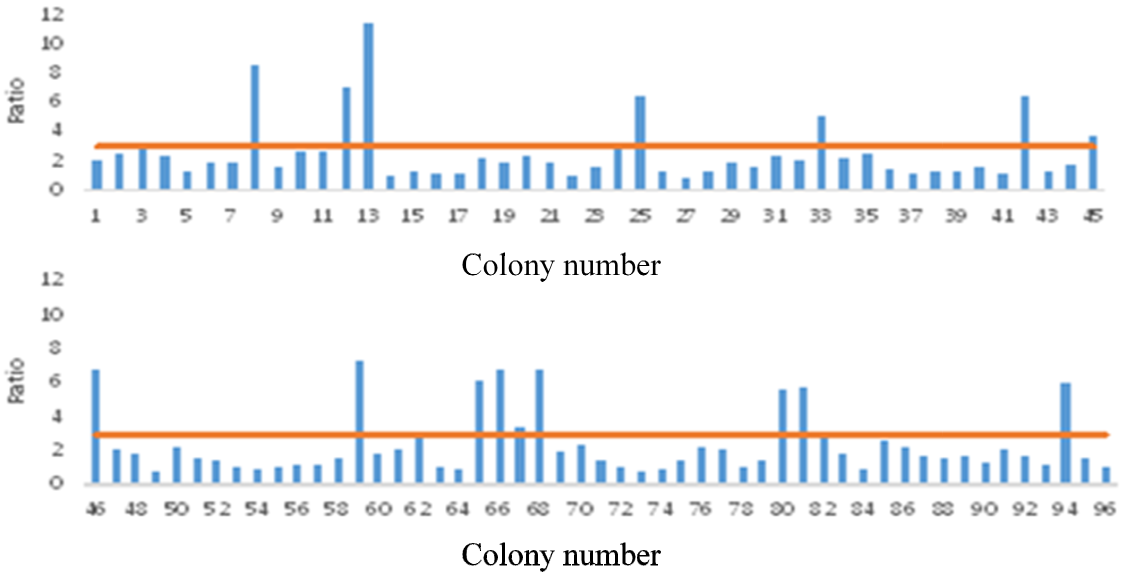

Figure B in S2 File. Analysis of clones for specific nanobodies directed against flagellin. A periplasmic extract of *E. coli* TG1 transformants, obtained after the second panning on purified flagellin, was used in ELISA to identify nanobodies binding the flagellins. Only nanobodies for which the ELISA ratio between flagellin-coated wells and non-coated wells was at least 3 were further analysed. The calculated ratio is on the y axis and the x axis represents the colony number. The orange line represents the ratio of 3.

If the ratio was equal or larger than three, the nanobody-encoding genes from the positive clones were amplified from the pHEN4 vector using the primers MP57 (5'-TTATGC TTCCGGCTCGTATG-3') and GIII (5'-CCACAGACAGCCCTCATAG-3') and sequenced. Nucleotide sequences were obtained for 13 clones (clones Nb2Flag8, Nb2Flag24, Nb2Flag25, Nb2Flag33, Nb2Flag42, Nb2Flag45, Nb2Flag46, Nb2Flag59, Nb2Flag66, Nb2Flag67, Nb2Flag80, Nb2Flag81 and Nb2Flag82) and these were aligned.

The nanobodies were grouped into three sequence families, on the basis of the CDR3 sequences of the 13 clones (Figure C in S2 File).

```
             ←----------FR1----------→ CDR1  ←-------FR2-----→ CDR2   ←------------------FR3-------------→     CDR3         ←---FR4---→
Nb2Flag8     QVQLQESGGGLVQPGGSLRLSCVGPASILIMNPMGWYRQAPGKQREYVASITTIGSTD-YVDSVKGRFIISTDGA--TLNLQMNSLKPEDAGVYYCNTVPPSRAGNY-----WGQGTQVTVSS
Nb2Flag25    QVQLQESGGGLVQPGGSLKLSCVGPASIFVMSPMGWFRQAPGKQREYVASITTIGSTN-YVDSVKGRFIISTDGA--TLYLQMNSLKPEDAGVYYCNTVPPSRSGNY-----WGQGTQVTVSS
Nb2Flag59    QVQLQESGGGLVQPGGSLKLSCVGPASIFVMNPMGWFRQAPGKQREYVASITTIGSTN-YVDSVKGRFIISTDGA--TLYLQMNSLKPEDAGVYYCNTVPPSRSGNY-----WGQGTQVTVSS
Nb2Flag66    QVQLQESGGGLVQPGGSLKLSCVGPASIFVMNPMGWFRQAPGKQREYVASITTIGSTN-YVDSVKGRFIISTDGA--TLYLQMNSLKPEDAGVYYCNTVPPSRSGNY-----WGQGTQVTVSS
Nb2Flag33    QVQLQESGGGLVQPGGSLRLSCVASASILSRNAMGWYRQAPGKQREWVASITTIGSTD-YVDSVKGRFIMSRDGAKNTLYLQMNSLKPEDTALYYCNTVPPSRSGGY-----WGQGTQVTVSS
Nb2Flag46    QVQLQESGGGLVQPGGSLRLSCVASASILSRNAMGWYRQAPGKQREWVASITTIGSTD-YVDSVKGRFIMSRDGAKNTLYLQMNSLKPEDTALYYCNTVPPSRSGGY-----WGQGTQVTVSS
Nb2Flag81    QVQLQESGGGLVQPGGSLRLSCVASASILSRNAMGWYRQAPGKQREWVASITTIGSTD-YVDSVKGRFIMSRDGAKNTLYLQMNSLKPEDTALYYCNTVPPSRSGGY-----WGQGTQVTVSS
Nb2Flag82    QVQLQESGGGLVQPGGSLRLSCVASASILSRNAMGWYRQAPGKQREWVASITTIGSTD-YVDSVKGRFIMSRDGAKNTLYLQMNSLKPEDTALYYCNTVPPSRSGGY-----WGQGTQVTVSS

Nb2Flag24    QVQLQESGGGLVQAGDSLRLSCAASGDTFSRYAMGWFRQAPGKEREFVAAITGDGRTTAYADSVKGRCTISRDNAKNTVNLQMNSLKPEDTAVYYCAASRQYYYTDYKHWESWGQGTQVTVSS
Nb2Flag42    QVQLQESGGGLVQAGDSLRLSCAASGDTFSRYAMGWFRQAPGKERDFVAAITGNGRTTAYADSVKGRCTISRDNAKNTVNLQMNSLKPEDTAVYYCAAIRQYYYTDYKHWESWGQGTQVTVSS

Nb2Flag67    QVQLQESGGGLVQAGGSLRLSCTASGRAFSAYAMAWFRQAPGKEREFVAAINRSGGRPYYVDSVKGRFTISRDSAKSTVDLQMSSLKPEDTAVYYCAVGIRPPLYDF-----WGQGTQVTVSS
Nb2Flag45    QVQLQESGGGLVQPGGSLRLSCTASGRAFSAYAMAWFRQAPGKEREFVAAINRSGGRPYYVDSVKGRFTISRDSAKSTVDLQMSSLKPEDTAVYYCAVGIRPPLYDF-----WGQGTQVTVSS
Nb2Flag80    QVQLQESGGGLVQPGGSLRVSCAASGRAFSAYAMAWFRQAPGKEREFVAAINRSGGRPYYVDSVKGRFTISRDSAKSTVDLQMSSLKPEDTAVYYCAVGIRPPLYDF-----WGQGTQVTVSS
```

Figure C in S2 File. Nanobodies alignment and grouping into three sequence families based on their CDR3 sequences.

To introduce a C-terminal histidine tag (His-tag), the nanobodies Nb2Flag8, Nb2Flag24 and Nb2Flag67 were cloned in the pHEN6c vector (Serge Muyldermans, personal communication), a derivative of the pHEN6 vector (Conrath *et al.*, 2001).

The nanobody sequences were PCR-amplified using the In-Fusion primers IF-NB1 (5′-TGGCCCAGGTGC AGCTGCAGGAGTCTGGAG-3′ carrying the PstI site) and IF-NB2 (5′-TGAGGAGAC GGTGACCTGGGTCC-3′ carrying the BstEII site). The PCR fragments were introduced in the pHEN6c, digested with PstI and BstEII, using the In-Fusion® HD Cloning Kit (Takara Bio USA, Inc). The resulting constructs were transformed into $CaCl_2$-competent *E. coli* DH5α (Dagert and Ehrlich, 1979) and transformants were selected on LB-agar plates supplemented with 100 µg/ml carbenicillin. Colonies were screened by PCR with the primers FP24 (5′-CGCCAGGGTTTTCCCAGTCACGAC-3′) and RP24 (5′–AGCGGATAACAATTT CACACAGGA-3′). PCR-positive colonies were sequenced to confirm that the nanobodies were correct and carried the C-terminal His-tag.

Footnote:

The pHEN6 vector is equivalent to the pHEN4 vector, except that the hemagglutinin tag and gene III were replaced by a His-tag to allow detection and purification (Conrath *et al.*, 2001). However, cloning of VHH genes in the pHEN6 resulted in an out of reading frame His-tag. The pHEN6c (Serge Muyldermans, personal communication) is a derivative of pHEN6 in which the reading frame with the His-tag is restored. Both plasmids pHEN6 and pHEN6c carry the ampicillin resistance gene.

The pHEN6c (with lower case "c") is different from the pHEN6C (with capital "C") described in Conrath *et al.* (2011). The pHEN6C is the pHEN6 with a chloramphenicol resistance gene instead of the ampicillin resistance gene.

References

Arbabi Ghahroudi M., Desmyter A., Wyns L., Hamers R. & Muyldermans S. (1997). Selection and identification of single domain antibody fragments from camel heavy-chain antibodies. *FEBS Lett.* 414: 521–526.

Conrath, K.E., Lauwereys, M. Galleni, M., Matagne, A., Frère, J.-M., Kinne, J., Wyns, L. & Muyldermans, S. (2001). β-lactamase inhibitors derived from single-domain antibody fragments elicited in the *Camelidae*. *Antimicrob. Agents Chemother.* 45: 2807-2812.

Dagert, M. & Ehrlich, S. D. (1979). Prolonged incubation in calcium chloride improves the competence of *Escherichia coli* cells. *Gene* 6: 23-38.

Pardon, E., Laeremans, T., Triest, S., Rasmussen, S.G.F., Wohlkönig, A., Ruf, A., Muyldermans, S., Hol, W.G.J., Kobilka, B.K. & Steyaert, J. (2014). A general protocol for the generation of Nanobodies for structural biology. *Nature Protocols* 9: 674-693.
