# Supplementary material for: In planta expression of nanobody-based designer chicken antibodies targeting Campylobacter
Source: PLoS One. 2018 Sep 27;13(9):e0204222. doi: 10.1371/journal.pone.0204222 (PMC6160005; doi:10.1371/journal.pone.0204222)
Supplement: S3 File — (PDF) [file pone.0204222.s003.pdf]

S3 File: Thermal and pH stability of nanobodies

The Thermofluor assay, also known as Differential Scanning Fluorimetry or Protein Thermal Shift Assay, was used to determine the stability of the nanobodies under different pH conditions using SYPRO orange dye (Sigma-Aldrich cat. no. S5692). The SYPRO orange dye undergoes a significant increase in quantum yield upon binding hydrophobic environments that become exposed during protein denaturation, allowing sensitive detection by fluorescence spectroscopy (Hawe *et al.*, 2008). SYPRO orange dye has excitation/emission wavelengths (λex 470 nm / λem 570 nm) that are compatible with filter sets found on real-time PCR instruments allowing their adaptation for protein thermal denaturation assays (Niesen *et al.*, 2007). The real-time PCR instrument used in this assay was the CFX Connect Real-Time PCR Detection System (BIO-RAD, cat. no. 1855201).

This assay allowed us to monitor the changes in protein folding under different pH conditions (96 different conditions of the Durham pH Screen MD1-101, Molecular Dimensions) through a systematic increase in temperature and the simultaneous measurement of the fluorescence emission of the dye, which undergoes a significant increase in quantum yield upon binding hydrophobic regions in denatured proteins (Huynh and Partch, 2015).

Method

Firstly, the protein concentration and SYPRO orange dye ratio were optimized for best assay performance. For this purpose, different concentrations of nanobody (0.1 mg/ml, 0.2 mg/ml and 0.5 mg/ml) were tested in the presence of 1x, 2x, 5x and 10x SYPRO orange dye concentration, respectively.

A total volume of 20 µl of a nanobody solution at the selected concentration in PBS buffer and SYPRO orange dye at the selected dilution was used in a 96 well plate. After preparing the plate, it was centrifuged for 2 minutes at 4000 rpm at 4°C, and it was placed inside a CFX Connect Real-Time PCR Detection System to perform the thermal denaturation assay. CFX Connect Manager 3.1 was used to run a melting curve protocol using a temperature gradient of 25-90°C, increasing 1°C per minute, with a fluorescence reading taken in every well at every temperature increment, using the FRET setting fluorophore (455-485 nm wavelength range for excitation and 567-596 nm for the collection of the emission).

The optimal protein concentration/dye ratios were determined for nanobodies Nb2Flag8, Nb2Flag24, Nb2Flag67, Nb5 and Nb23 and they were used to perform the Durham pH Screen according to the manufacturer instructions. In brief, 10 µl of each screen condition were transferred into the corresponding well of a 96-wells plate and 10 µl of a solution of the

nanobody and dye at double of the appropriate concentration/dye dilution were added to each well. After the plate was prepared and sealed, it was centrifuged for 2 minutes at 4000 rpm at 4°C, and it was placed inside a CFX Connect Real-Time PCR Detection System to perform the thermal denaturation assay, using the same settings as described above. All the data was exported in ".csv" format and analyzed using GraphPad Prism 7.0.

Results

Nanobodies Nb5, Nb2Flag8 and Nb2Flag67 are showing a similar stability for the complete pH range. The reference T_m value for Nb5 in PBS buffer is 67.3°C with a temperature variation across the pH range of less than 5.4°C, with the lowest T_m value of 61.9°C at pH 10.9. In the case of Nb2Flag8, the T_m in PBS was 60.5°C, while reaching the lowest T_m at pH 4.1 of 51.4°C, which represents a decrease of the 15% relative to the reference temperature in PBS. Nb2Flag67 has a T_m in PBS of 64.3°C and the lowest value at pH 10.9 of 55.8°C, for a maximum variation in temperature in the pH range of 13 %. Melting curves were only obtained for nanobody Nb2Flag23 between pH 6.6 and 10.9. The T_m values calculated for all the conditions in this range are between 56.7°C at pH 6.6 and 70.6°C at pH 9.7, which is close to the reference T_m in PBS.

It is known that not all globular folded proteins show ideal profiles in this assay, which hinders the analysis of their stability by this method. Several causes have been proposed for this phenomenon, most of which are related to the specific biophysical features of the particular protein (Huynh and Partch, 2015). This shortcoming of the technique prevented us from assessing the pH stability of Nb23 below pH 6.6. For NbFlag24 we could not obtain data for all pH conditions tested.

Figure A in S3 File shows the plots of the T_m vs a selection of solution conditions covering a pH range from 4.1 to 10.9. Automated processing of thermal denaturation curves was used to truncate the dataset in order to remove post-peak quenching of the fluorescence signal, according to the macro described in the supplementary material of Huynh and Partch (2015). The resulting sigmoidal curves are then fitted to a Boltzmann Equation to determine the T_m values.

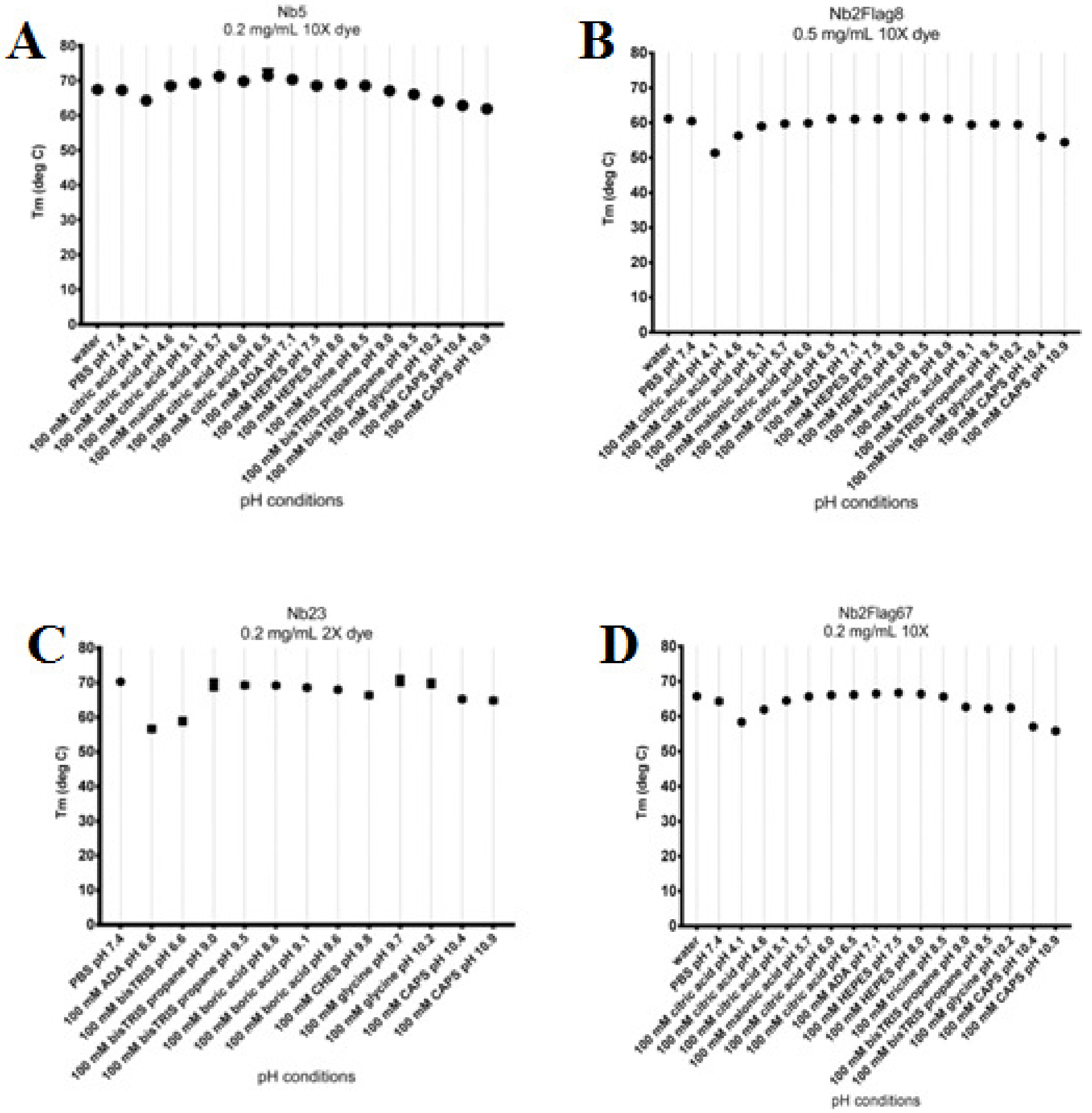

Figure A in S3 File. Melting temperatures (T_m) *vs* pH conditions for **A**) 0.2 mg/ml of nanobody Nb5 combined with 10x SYPRO orange dye, **B**) 0.5 mg/ml of Nb2Flag8 and 10x dye **C**) 0.2 mg/ml of Nb23 and 2x dye and **D**) 0.2 mg/ml of Nb2Flag67 combined with 10x dye. T_m values at different pH conditions were obtained via thermal shift assays using the Durham pH Screen (Molecular Dimensions). Non-linear fitting to a Boltzmann Equation was applied to the sigmoidal curves to determine the T_m for each condition.

References

Hawe, A, Sutter, M, & Jiskoot, W. (2008). Extrinsic fluorescent dyes as tools for protein characterization. Pharm Res. 25: 1487–1499.

Huynh, K., & Partch, C.L. (2015). Analysis of protein stability and ligand interactions by Thermal Shift Assay: Protein stability by Thermal Shift Assay. In Current Protocols in Protein Science, J.E. Coligan, B.M. Dunn, D.W. Speicher, and P.T. Wingfield, eds. (Hoboken, NJ, USA: John Wiley & Sons, Inc.), pp. 28.9.1-28.9.14.

Niesen, F, Berglund, H, & Vedadi, M. (2007). The use of differential scanning fluorimetry to detect ligand interactions that promote protein stability. Nature protocols 2: 2212–2221.
