# Supplementary material for: In planta expression of nanobody-based designer chicken antibodies targeting Campylobacter
Source: PLoS One. 2018 Sep 27;13(9):e0204222. doi: 10.1371/journal.pone.0204222 (PMC6160005; doi:10.1371/journal.pone.0204222)
Supplement: S4 File — (PDF) [file pone.0204222.s004.pdf]

S4 File: Construction of the synthetic Nb-IgY or Nb-IgA fusion genes

Synthetic fusion genes (Figure A in S4 File) were made in which the Nb5 sequences were fused to the Fc-domain of chicken IgA (Mansikka, 1992) or the constant domains of chicken IgY (Parvari *et al.*, 1988). The codon usage of the IgA and IgY domains was optimized for expression in *A. thaliana*.

In the synthetic gene constructs the Nb5-encoding sequence was preceded by the signal sequence of the seed storage protein 2S2 of *A. thaliana* for targeting to the endoplasmic reticulum. At the C-terminus of the IgY or IgA domains, a Histidine-tag and a KDEL signal were added. The His-tag was introduced since chicken IgY or IgA cannot be purified with the standard immunoglobulin-binding protein Protein A- or Protein G-agarose. The His-tag would facilitate the detection in western blot and ELISA, and also the purification of the in plant- or in seed-produced Nb fusions. Unfortunately the introduced His-tag could only be detected after denaturation of the Nb fusions in western blot. The KDEL sequence is needed for retention in the lumen of the endoplasmic reticulum. The attachment sites (attB1 and attB2), flanking the synthetic Nb-Ig fusion genes, allow the cloning of the synthetic genes in the pDONR221 Gateway donor vector (Gateway Technology) yielding in entry clones pGV5701 (Figure B in S4 File) and pGV5702 carrying the Nb5-IgA or the Nb5-IgY (Figure B in S4 File) fusion gene, respectively.

The other nanobodies (Nb23, Nb2Flag8, Nb2Flag23, Nb2Flag67 and nanobody V1 directed against the F4-fimbriae of enterotoxigenic *E. coli* (Virdi *et al*, 2013)) were introduced in the entry clones carrying the Nb5-IgA or Nb5-IgY fusion by exchanging the Nb5 sequence by the other Nb sequences. The Nb5 was removed by restriction of the entry clones with PstI and BstEII and replaced by the PCR-amplified nanobody-encoding sequences of Nb23, Nb2Flag8, Nb2Flag24, Nb2Flag67 and the nanobody V1 (positive control) using the primers T-NbS1 (ACGCCCAGGTGCAGCTGCAAGAGTCTGGAGGAGGCTTGGTGCAG, PstI site is underlined) and T-NbS2 (TGAGGAGACGGTGACCTGGGTC, BstEII site is underlined).

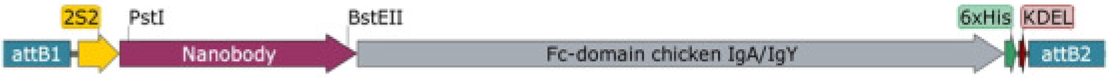

Figure A in S4 File. Construction of chimeric antibodies for *in planta* expression. The synthetic genes were introduced into the pDONR221 vector using the Gateway technology via the attB1 and attB2 sites. The 2S2 signal peptide of the *Arabidopsis* 2S2 seed storage protein was added for translocation to the endoplasmic reticulum and the C-terminal KDEL retention in the endoplasmic reticulum. The His-tag facilitates detection in western blot.

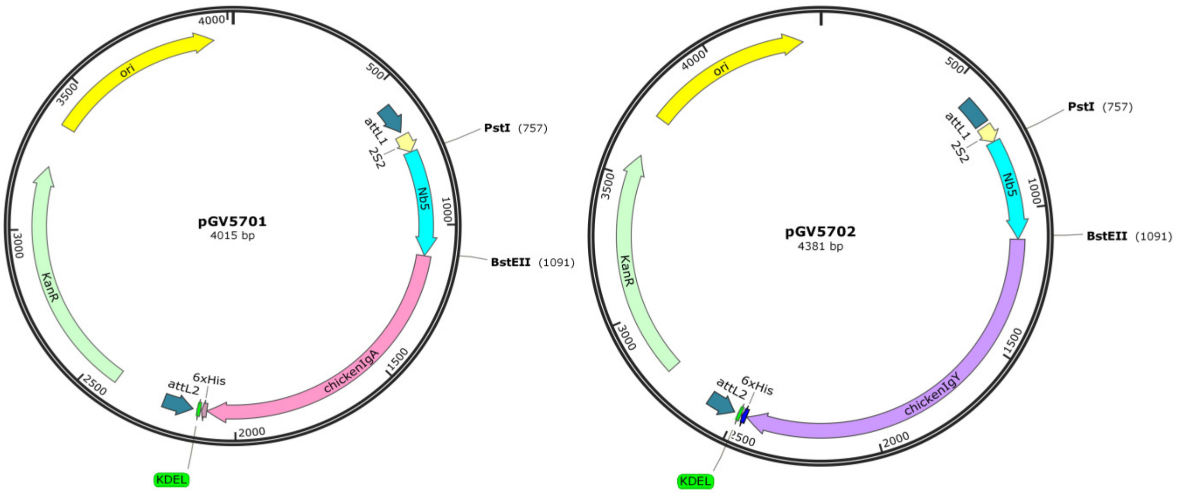

Figure B in S4 File. The entry clones pGV5701 and pGV5702 carrying respectively the synthetic fusion genes Nb5-IgAand Nb5-IgY cloned in pDONR221.

After the exchange of Nb5 by the other Nbs, the resulting entry clones in pDONR221 were sequenced. The plasmid DNAs from clones with a correct sequence were isolated with the Qiagen miniprep DNA extraction kit. The miniprep DNA carrying the Nb-IgY or Nb-IgA gene fusions were introduced via an LR reaction in the Gateway-compatible vector pEAQ-*HT*-DEST1 (Sainsbury *et al.*, 2009) for transient expression in *N. benthamiana* leaves (De Buck *et al.*, 2012) or in the Gateway-compatible vector pPhasGW (Morandini *et al.*, 2011) for stable expression in *A. thaliana* seeds. In the pPhasGW the Nb-Ig fusions are under control of the strong seed-specific β-phaseolin promoter. The T-DNA also encodes the kanamycin resistance gene *nptII* for the selection of transgenic plants.

The Nb-Ig gene fusions in the pEAQ-*HT*-DEST1 vector were transformed in *Agrobacterium tumefaciens* strain LBA LBA4404 (McCormac *et al.*, 1998) via electroporation. Also the Nb-Ig fusions in the pPhasGW vector were transformed in *A. tumefaciens* C58C1 Rif^{R} (pMP90) via electroporation. The transformed C58C1 Rif^{R} (pMP90) carrying the Nb-Ig fusions in pPhasGW were used to transform *A. thaliana* ecotype Columbia (Col-0) by the floral dip technique (Clough and Bent, 1998)

References

Clough, S. J., and Bent, A. F. (1998). Floral dip: a simplified method for *Agrobacterium*-mediated transformation of *Arabidopsis thaliana*. Plant J. 16: 735-743.

De Buck, S., Virdi, V., De Meyer, T., De Wilde, K., Piron, R., Nolf, J., *et al.* (2012). Production of camel-like antibodies in plants. *Methods Mol Biol*. 911:305–324.

Mansikka, A. (1992). Chicken IgA H chains. Implications concerning the evolution of H chain genes. *J. Immunol*. 149: 855-861.

McCormac, A.C., Elliott, M.C., & Chen, D.F. (1998). A simple method for the production of highly competent cells of *Agrobacterium* for transformation via electroporation. *Mol Biotechnol* 9: 155-159.

Morandini F, Avesani L, Bortesi L, Van Droogenbroeck B, De Wilde K, Arcalis E, et al. (2011). Non-food/feed seeds as biofactories for the high-yield production of recombinant pharmaceuticals. *Plant Biotechnol J*. 9: 911–921.

Parvari, R., Avivi, A., Lentner, F., Ziv, E., Tel-Or, S., Burstein, Y., & Schechter, I. (1988). Chicken immunoglobulin gamma-heavy chains: limited VH gene repertoire, combinatorial diversification by D gene segments and evolution of the heavy chain locus. *EMBO J.* 7: 739-744.

Sainsbury F, Thuenemann EC, & Lomonossoff GP. (2009). pEAQ: versatile expression vectors for easy and quick transient expression of heterologous proteins in plants. *Plant Biotechnol J.* 7: 682–693

Virdi, V., Coddens, A., De Buck, S., Millet, S., Goddeeris, B.M., Cox, E., De Greve, H., & Depicker, A. (2013). Orally fed seeds producing designer IgAs protect weaned piglets against enterotoxigenic *Escherichia coli* infection. *Proc Natl Acad Sci USA* 110: 11809–11814.
