# Supplementary figures and images for: In planta expression of nanobody-based designer chicken antibodies targeting Campylobacter
Source: PLoS One. 2018 Sep 27;13(9):e0204222. doi: 10.1371/journal.pone.0204222 (PMC6160005; doi:10.1371/journal.pone.0204222)

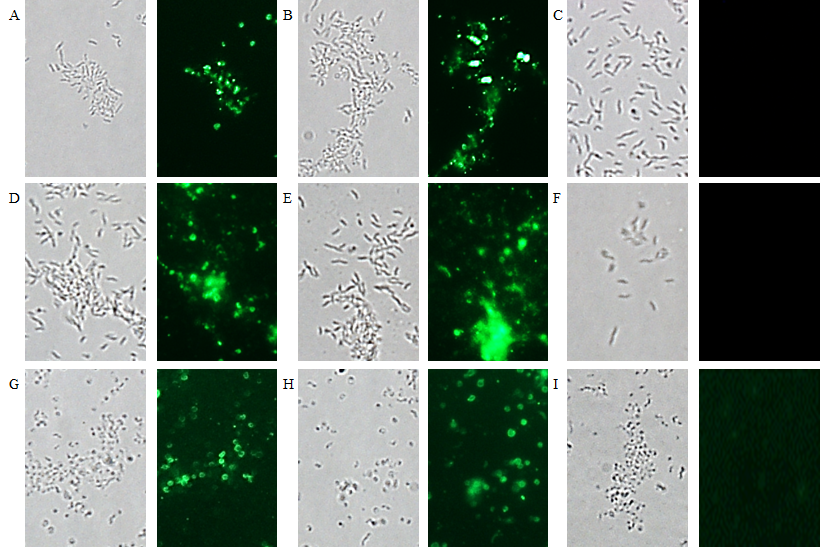

Supplement: S1 Fig — Immunofluorescence microscopy confirms the interaction of Nb5 and Nb23 with Campylobacter isolates. (A, B, C) C. jejuni strain KC40, (D, E, F) C. jejuni strain Cam12/0156 and (G, H, I) C. coli strain K43/5. The binding of Nb5 is shown in A, D and G and the binding of Nb23 in B, E and H. As a negative control, the fluorescently labelled (C, F, I) V1 nanobody was used. (TIF) [file pone.0204222.s005.tif]

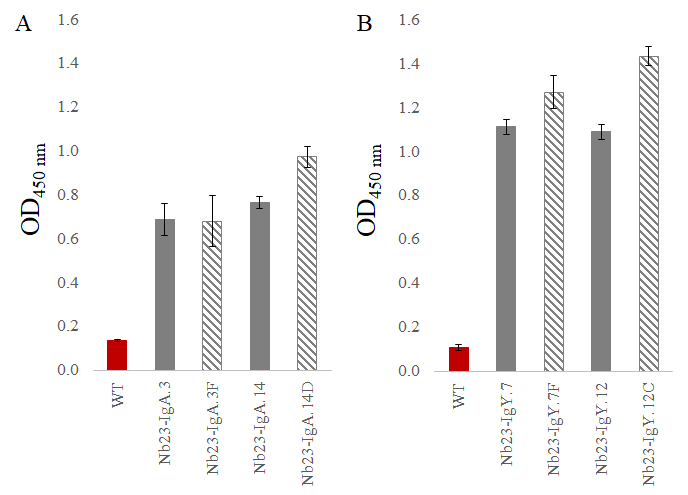

Supplement: S2 Fig — ELISA was used for the analysis of seed extracts from A. thaliana plants transformed with (A) Nb23-IgA constructs and (B) Nb23-IgY constructs. The results of the extracts of the homozygous plants are visualised by the histogram with hatched shading. Extract of wild-type A. thaliana seeds was used as a negative control. The ELISA was developed using anti-IgA or anti-IgY conjugated to HRP. The error bars correspond to the standard deviation. (TIF) [file pone.0204222.s006.tif]

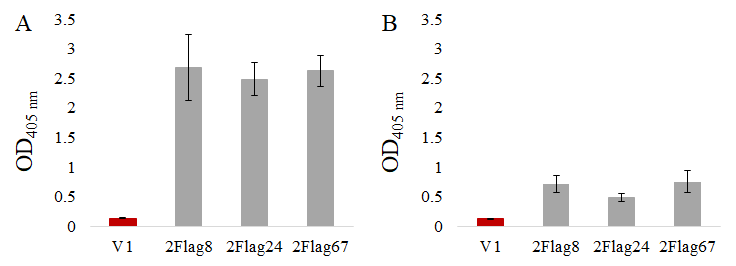

Supplement: S3 Fig — ELISA for the confirmation of the interaction of anti-flagellin nanobodies with (A) purified flagellins and (B) C. jejuni KC40. Bound His-tagged nanobodies were detected with mouse anti-histidine monoclonal antibodies and goat anti-mouse IgG. The error bars correspond to the standard deviation. (TIF) [file pone.0204222.s007.tif]
