# Supplementary material for: In planta expression of nanobody-based designer chicken antibodies targeting Campylobacter
Source: PLoS One. 2018 Sep 27;13(9):e0204222. doi: 10.1371/journal.pone.0204222 (PMC6160005; doi:10.1371/journal.pone.0204222)
Supplement: S1 Table — (PDF) [file pone.0204222.s008.pdf]

S1 Table. Determination of the chimeric antibody concentration in extracts of *A. thaliana* seeds, with the Chicken IgA ELISA Kit (Abcam; Product code ab157691).

Chimeric antibody	Concentration (µg/ml) ± SD	µg/mg seed ± SD	%TSP ± SD
Nb2Flag8-IgA	72.12 ± 5.95	7.21 ± 0.60	3.61 ± 0.30
Nb2Flag24-IgA	106.19 ± 6.27	10.62 ± 0.63	5.31 ± 0.32
Nb2Flag67-IgA	47.54 ± 0.89	4.75± 0.09	2.38 ± 0.05
